# Paeoniflorin Alleviates Oxygen–Glucose Deprivation/Reoxygenation Injury by Mediating Crosstalk Between Neurons and Endothelial Cells Through the VEGF/PI3K-AKT/mTOR Pathway

**DOI:** 10.3390/ph19091339

**Published:** 2026-08-24

**Authors:** Zike Xu, Hongxia Luo, Yimin Zhao, Xuhui Wang, Sha Chen

**Affiliations:** 1 Key Laboratory of Biochemistry and Molecular Pharmacology, Department of Pharmacology, Chongqing Medical University, Chongqing 400016, China; 2022222622@stu.cqmu.edu.cn (Z.X.); 2022222586@stu.cqmu.edu.cn (H.L.); 2021222661@stu.cqmu.edu.cn (Y.Z.); 2 Department of Neurosurgery, The Second Afffliated Hospital of Chongqing Medical University, 76 Linjiang Road, Chongqing 400010, China

**Keywords:** cerebral ischemia–reperfusion injury, paeoniflorin, VEGF/PI3K-AKT/mTOR pathway, network pharmacology, neuroprotection, crosstalk

## Abstract

**Background/Objectives**: Cerebral ischemia–reperfusion injury (CIRI) poses therapeutic challenges because of oxidative stress, blood–brain barrier disruption, and neuronal apoptosis, limiting current treatments. Paeoniflorin (PF) from *Paeonia lactiflora* has neuroprotective potential, but its multi-target mechanisms remain unclear. This study investigated the role and mechanisms of PF in CIRI, focusing on neuron–endothelial crosstalk. **Methods**: Oxygen–glucose deprivation/reoxygenation (OGD/R) models were established using SH-SY5Y (human neuroblastoma) and HCMEC/D3 cells (human cerebral microvascular endothelial). Network pharmacology was used to predict potential PF targets and pathways. RNA sequencing, molecular docking, and molecular dynamics simulation were performed to screen and evaluate PF binding characteristics with key targets, and MTT, flow cytometry, Western blotting, and co-cultures were employed to detect paracrine interactions. **Results**: Network pharmacology and transcriptomics identified VEGF/PI3K-AKT/mTOR pathway enrichment. Molecular docking confirmed stable PF binding to VEGF-A (−8.4 kcal/mol), AKT (−5.5 kcal/mol), and mTOR (−9.6 kcal/mol). PF (10–80 μM) showed no cytotoxicity and reduced OGD/R injury in a concentration-dependent manner (maximal at 40 μM). PF activated VEGF/PI3K-AKT/mTOR signaling, reducing apoptosis by 57% (SH-SY5Y) and 33% (HCMEC/D3); PI3K inhibitor LY294002 abolished these effects. PF-treated HCMEC/D3-conditioned media enhanced OGD/R neuronal viability, verifying paracrine crosstalk. **Conclusions**: PF alleviated CIRI by directly protecting neurons and indirectly modulating neuron–endothelial crosstalk through VEGF/PI3K-AKT/mTOR activation, supporting its multi-target therapeutic potential.

## 1. Introduction

Cerebral ischemia–reperfusion injury (CIRI) is a critical pathological process that occurs following blood flow restoration in ischemic stroke, significantly contributing to the high global rates of incidence, disability, and mortality associated with this condition [1]. Many patients experience worsened cerebral inflammation and oxidative stress because of reperfusion injury, leading to poor prognoses [2,3]. Although reperfusion therapies such as intravenous thrombolysis and endovascular thrombectomy significantly improve blood flow, current clinical strategies, including thrombolytics, antiplatelet agents, neuroprotective drugs such as edaravone, and hypothermia, are constrained by narrow therapeutic windows, side effects, and variable efficacy [4,5].

Endothelial cell apoptosis and blood–brain barrier (BBB) [6] disruption propagate injury signals to neurons through specific molecular signaling networks. In maintaining endothelial cell survival and BBB integrity, the vascular endothelial growth factor (VEGF) pathway primarily promotes angiogenesis and improves blood perfusion by activating VEGF receptors, while also exerting direct neuronal neurotrophic and anti-apoptotic effects [7]. The PI3K-AKT/mTOR pathway serves as a critical intracellular survival signaling axis, inhibiting neuronal apoptosis [8] and promoting protein synthesis when activated [9]. These two pathways are closely interregulated; VEGF can synergistically enhance neuronal survival signals through PI3K-AKT [10] signaling activation, while Protein Kinase B (AKT) activation further upregulates VEGF [11,12] expression, forming a positive feedback loop [13]. Both pathways are suppressed in cerebral ischemia–reperfusion injury (CIRI), leading to exacerbated neuronal apoptosis and impaired synaptic function. Targeted VEGF and PI3K-AKT/mTOR signaling activation may synergistically attenuate reperfusion injury and confer neuroprotection [14,15]. However, the regulatory mechanisms underlying this interactive network in CIRI remain poorly elucidated, and validation using small molecules capable of simultaneously targeting this network is currently lacking.

Traditional Chinese herbal medicine contains a diverse array of bioactive constituents capable of modulating multiple biological pathways; this makes it highly compatible with the complex pathological processes of CIRI and an ideal candidate for investigating similarly multifactorial diseases. *Paeonia lactiflora* Pall. (*Ranunculaceae*) is a classical herb frequently employed in ancient prescriptions for stroke treatment (Zhongfeng). Its active constituent, paeoniflorin (PF), is a typical monoterpene glycoside extracted and isolated from its roots. PF possesses a broad spectrum of significant biological activities, including anti-inflammatory, antioxidant, immunomodulatory, and neuroprotective effects [16,17]. In recent years, its clinical application value has attracted considerable attention: multiple clinical and basic research studies have focused on its therapeutic potential in depression and cerebrovascular diseases. It has demonstrated potential efficacy in alleviating depressive symptoms, regulating the neuroinflammatory microenvironment, and improving vascular function. Combined administration of PF and calycosin-7-glucoside exhibits favorable neuroprotective effects in both MCAO/R rat and oxygen–glucose deprivation/reoxygenation (OGD/R) cell models, with its mechanism closely associated with PI3K/AKT activation [18,19,20]. These findings suggest that PF may not function as a simple pathway activator or inhibitor, but rather exhibits restorative modulatory properties. However, the aforementioned studies were not conducted in CIRI models; although the neuroprotective and vasculoprotective activities of PF have been independently validated, whether these dual effects are achieved through this network to mediate endothelial–neuronal signaling crosstalk remains unverified.

**Figure 1:**
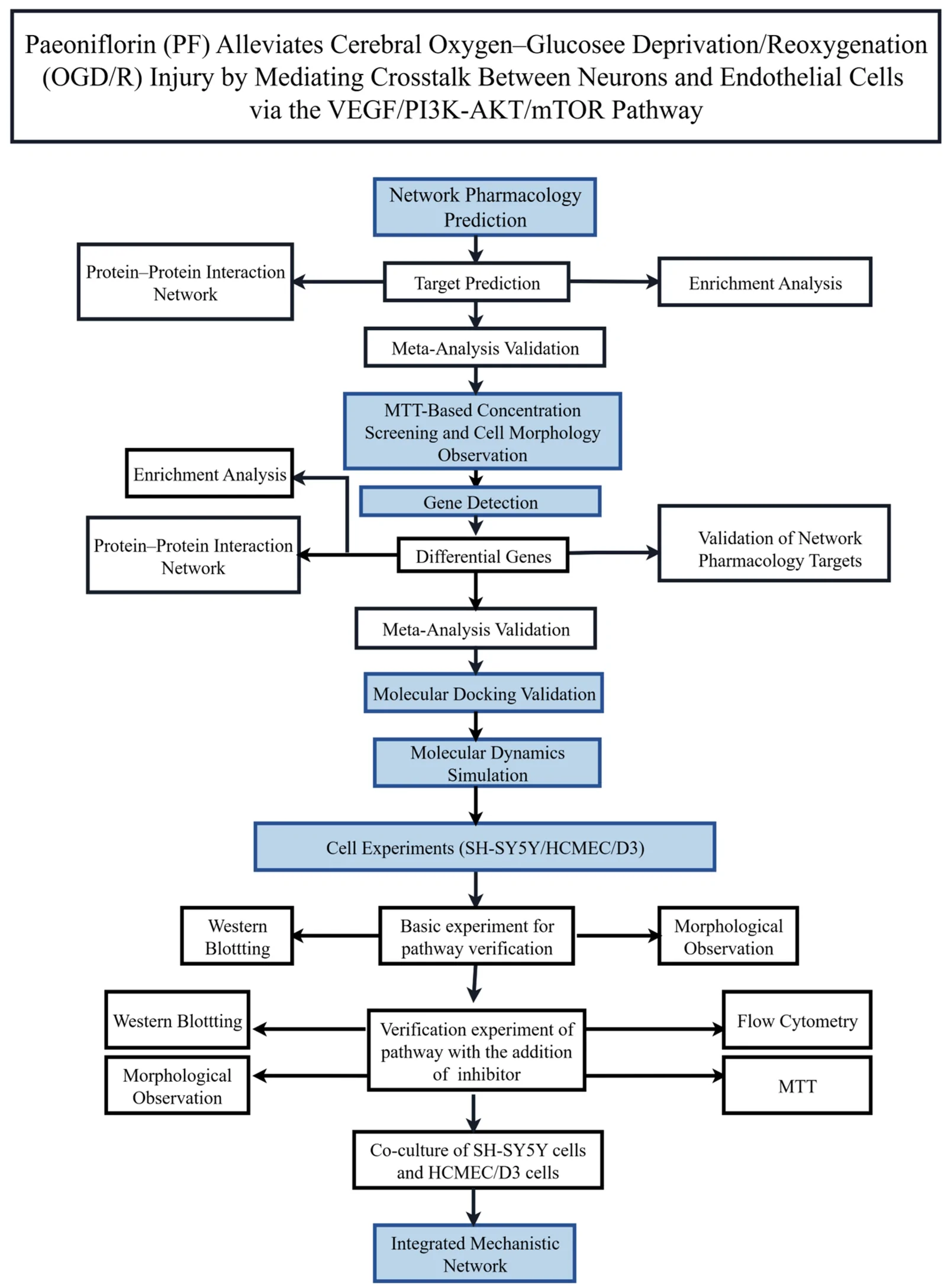
Research roadmap: Schematic diagram of the technical route for investigating PF alleviating cerebral ischemia–reperfusion injury through VEGF/PI3K-AKT/mTOR signaling (created in Figdraw).

It was hypothesized that during the pathological progression of CIRI, PF not only inhibits neuronal apoptosis by activating VEGF/PI3K-AKT/mTOR signaling, but also maintains endothelial cell survival and BBB structural integrity, mediating signal transduction between neurons and endothelial cells and supporting the cerebral vascular microenvironment. This study aimed to elucidate the specific molecular mechanisms by which PF regulates this interactive network, providing experimental evidence for the development of CIRI intervention strategies that simultaneously target both neural and vascular units. Research roadmap is illustrated in Figure 1.

## 2. Results

### 2.1. Network Pharmacology and Meta-Analysis Suggest That PF Prevents CIRI by Modulating Vascular Endothelial Cell Function Through the VEGF/PI3K-AKT Axis

**Figure 2:**
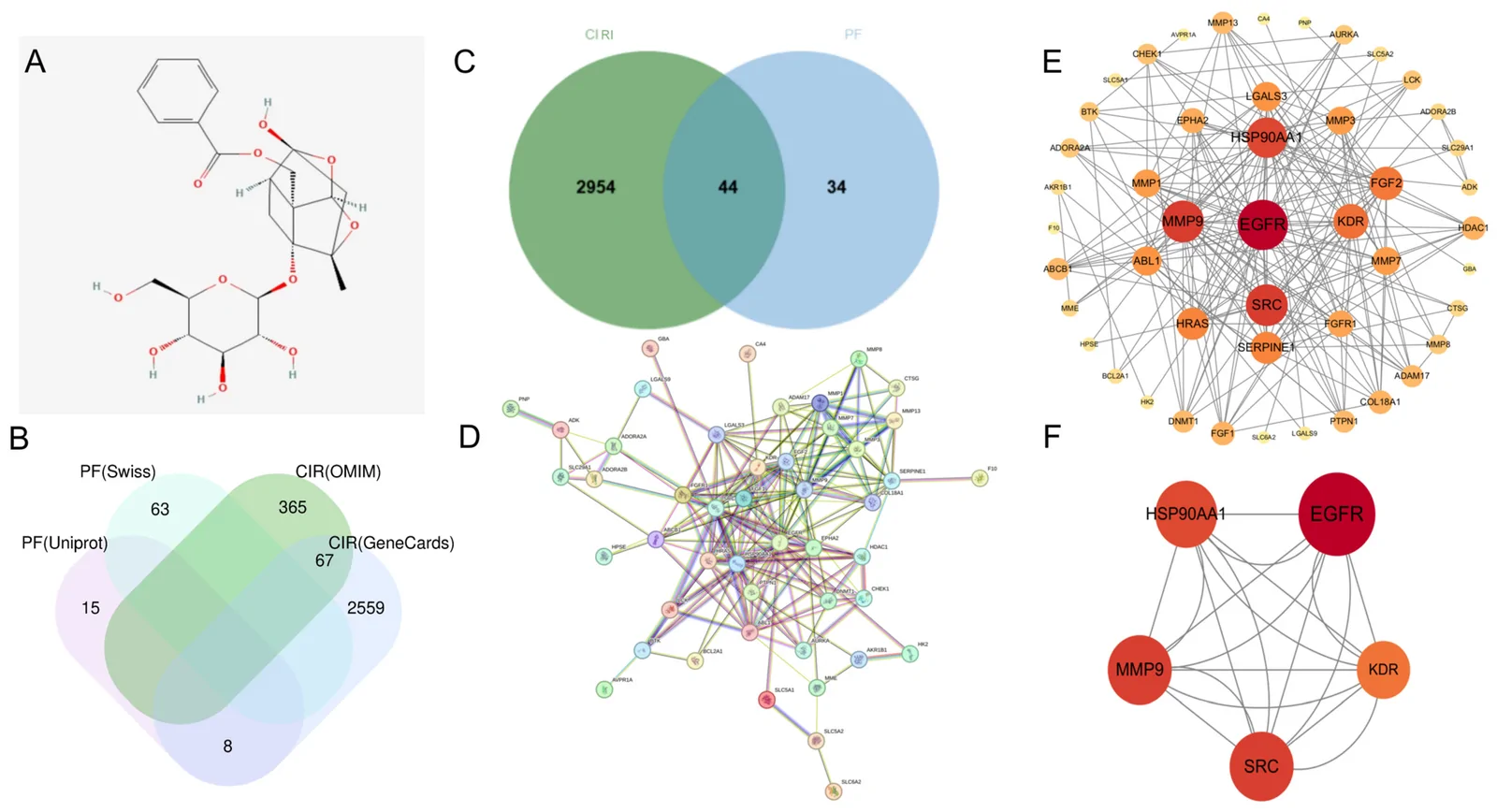
(**A**) 2D chemical structure of PF. (**B**) Intersection of potential PF targets (sourced from SwissTargetPrediction and UniProt) and cerebral ischemia–reperfusion (CIRI)-related genes (sourced from OMIM and GeneCards). (**C**) Common target genes of PF and CIRI. (**D**) The PPI network was constructed using STRING. Nodes denote proteins and edges denote interactions. Colors represent functional modules; sizes represent connectivity. (**E**) Protein–protein interaction (PPI) network of common targets constructed using STRING database. (**F**) Top five core targets identified in PF-CIRI PPI network using Cytoscape.

Target prediction was used to elucidate the pharmacological mechanisms by which PF exerts therapeutic effects against CIRI. The chemical structure of PF is depicted in Figure 2A. Putative target genes associated with PF and CIRI were retrieved from SwissTargetPrediction, Index-Calcnet-TargetNet, GeneCards, and OMIM (Figure 2B). Overlapping PF-related candidates from the first two databases yielded 78 genes; further intersection with CIRI-associated disease targets (*n* = 2998) narrowed these to 44 high-confidence targets (Figure 2C). A PPI network was constructed using STRING and analyzed by Cytoscape 3.9.1 to identify hub targets with the highest degrees of centrality, with EGFR (linked to PI3K activation), SRC (a PI3K-AKT regulator), and KDR (a VEGF pathway core node) all ranking in the top five (Figure 2D–F).

Gene Ontology (GO) and Kyoto Encyclopedia of Genes and Genomes (KEGG) enrichment analyses were performed to probe the underlying mechanisms involved. The results revealed coordinated endothelial function modulation, supporting our hypothesis that cerebroprotection by PF is mediated, at least partly, through the VEGF/PI3K-AKT endothelial regulatory axis.

**Figure 3:**
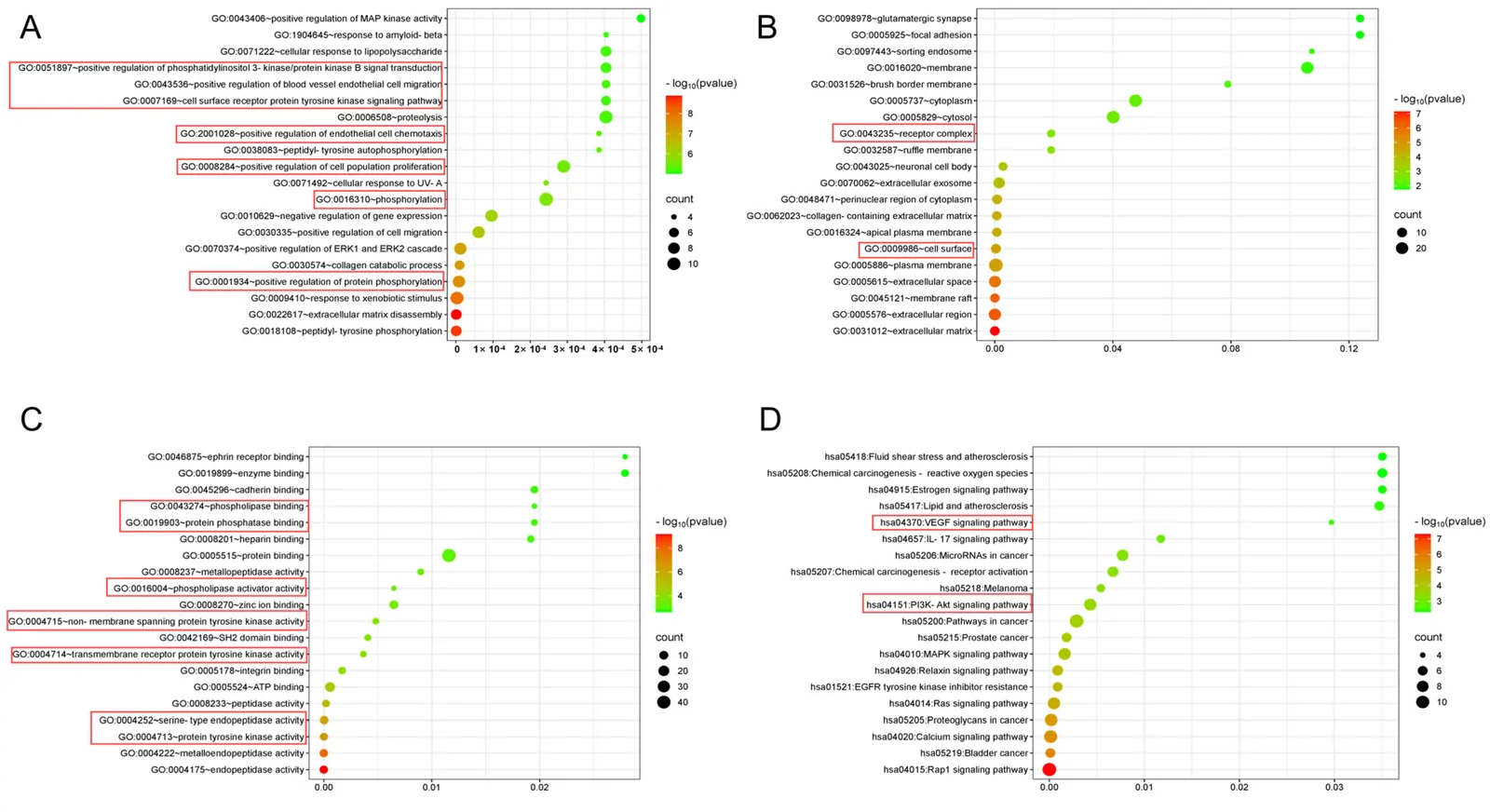
(**A**–**D**) Bubble plots of Gene Ontology (GO) enrichment analysis (biological processes—BPs; cellular components—CCs; molecular functions—MFs) and Kyoto Encyclopedia of Genes and Genomes (KEGG) pathway enrichment analysis for the common target genes.

GO enrichment across biological processes (BPs), cellular components (CCs), and molecular functions (MFs) provided complementary insights (Figure 3A–C). In BPs, the top terms (protein phosphorylation and its positive regulation, endothelial chemotaxis, and cell migration) suggest that PF enhances endothelial proliferative and migratory capacities, essential for microvasculature repair after CIRI. CC enrichment highlighted receptor complexes and cell surface compartments: membrane receptors such as KDR/VEGFR2 and EGFR initiate signaling, while cytoplasmic SRC translocates to the membrane upon activation, indicating that PF targets are centered on membrane-associated complexes and their intracellular hubs. MF enrichment showed significant binding activities to phospholipase and protein phosphatase, implying that PF may sustain pro-survival signaling through lipid second messengers and balanced phosphatase activity, counteracting oxidative stress and apoptosis.

KEGG analysis (Figure 3D) identified VEGF (hsa04370) and PI3K-AKT (hsa04151) as the top enriched pathways, which converge into a unified axis: VEGF/KDR recruits SRC, which activates PI3K-AKT, promoting angiogenesis and suppressing apoptosis—key effects for reversing CIRI pathology.

**Figure 4:**
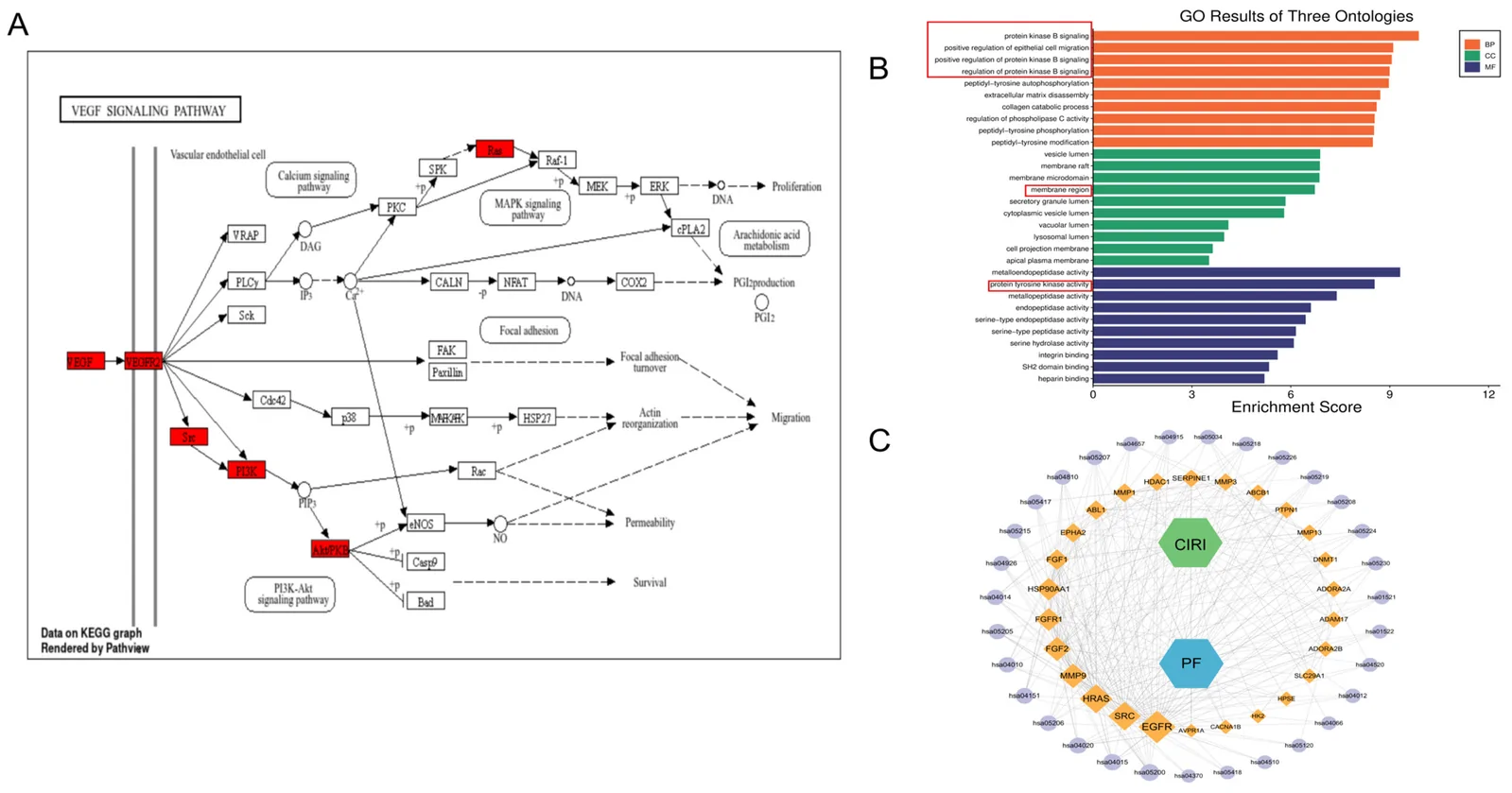
(**A**) Enrichment plot of common targets within the VEGF signaling pathway. (**B**) Bar chart of GO enrichment analysis results (BPs, CCs, MFs). (**C**) Network diagram illustrating the relationships among PF, CIR, targets, and pathways.

Integrated GO summary (Figure 4B) further consolidated these associations, with top processes including AKT signaling, endothelial migration, and membrane receptor signaling. The disease–target–pathway network (Figure 4C) highlighted these two pathways as major modules. Topological VEGF cascade mapping (Figure 4A) outlined a putative linear relay—VEGF/VEGFR2/SRC/PI3K-AKT—suggesting that PF may alleviate CIRI through this axis.

**Figure 5:**
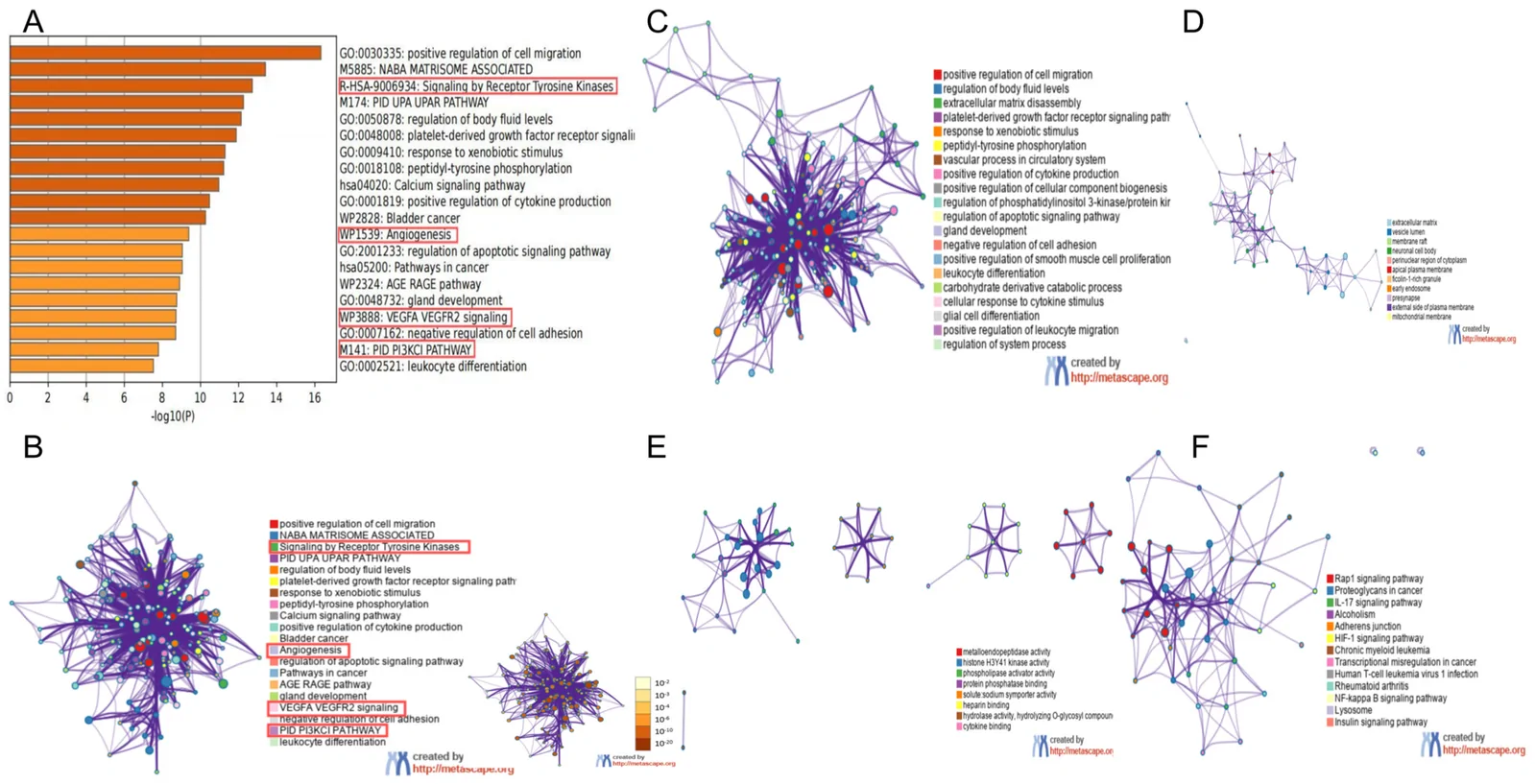
(**A**) Overview plot of meta-analysis enrichment. (**B**,**C**) Scatter plots of meta-analysis results (color intensity indicates significance level—light to dark: low to high). (**D**–**F**) Scatter plots of GO (BPs, CCs, MFs) and KEGG enrichment results derived from meta-analysis.

Metascape enrichment analysis (Figure 5A,B) revealed that PF-mediated protection against CIRI was predominantly due to enrichments in VEGF/VEGFR2 signaling, the PI3K-AKT axis, and angiogenesis-related tyrosine kinase receptors, aligning with our network pharmacology prediction centered on endothelial vascular protection.

Subsequent GO analysis of overlapping PF–CIRI targets using Metascape (Figure 5C) identified the top BPs, including positive regulation of cell migration, extracellular matrix (ECM) disassembly, PDGFR signaling, positive regulation of PI3K-AKT signaling, and positive regulation of cellular component biogenesis. These terms collectively indicate a pro-angiogenic and pro-survival functional profile, with potential anti-apoptotic effects in both neuronal and endothelial compartments. CC analysis (Figure 5D) localized PF-associated targets primarily to the ECM and plasma membrane rafts, where VEGFR2 and other receptor tyrosine kinases assemble, suggesting that PF may act through membrane receptor complexes to initiate downstream VEGF/PI3K-AKT cascades. MF enrichment (Figure 5E) revealed diverse activities, including metalloendopeptidase activity (implicated in ECM remodeling), phospholipase activator activity, and protein phosphatase binding (which may sustain PI3K-AKT activation), as well as histone kinase, ion transporter, and cytokine binding, indicating multi-modal endothelial homeostasis and inflammatory response regulation.

**Figure 6:**
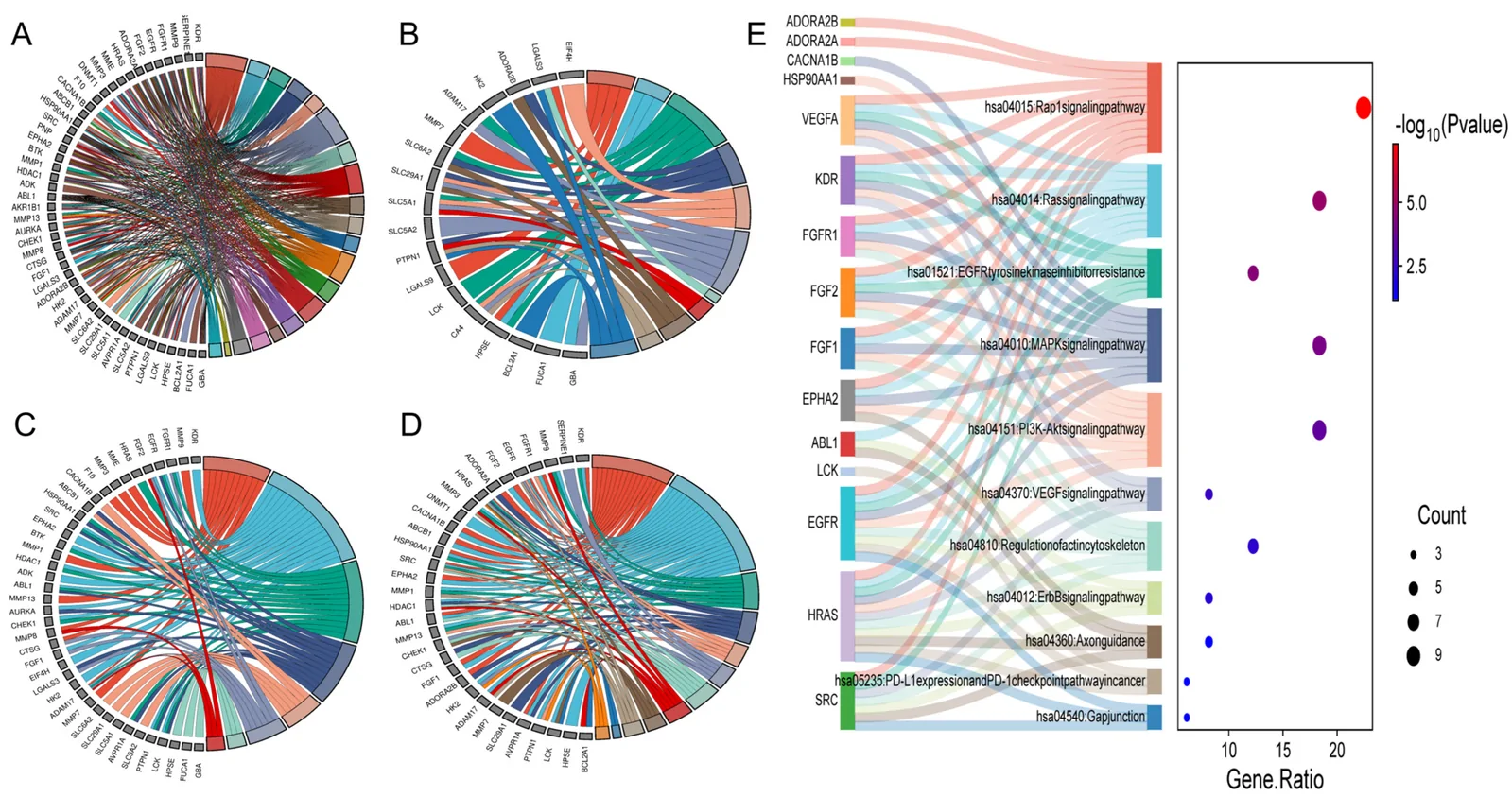
(**A**–**D**) Chord diagrams of GO (BPs, CCs, MFs) and KEGG enrichment results derived from meta-analysis. (**E**) Sankey bubble diagram illustrating PF-CIR-related targets and pathways based on meta-analysis.

KEGG pathway analysis (Figure 5F) further corroborated these findings, with secondary enrichment in HIF-1 signaling, along with cancer- and inflammation-related pathways that harbor MAPK/AP-1 and NF-κB signaling modules. HIF-1α upregulates VEGFA under hypoxia, whereas AP-1 and NF-κB may modulate proliferation and inflammatory responses, implying a coordinated network rather than isolated linear cascades. Chord diagrams (Figure 6A–D) and pathway–target mapping (Figure 6E) visually confirmed that core hub genes (VEGFA, EGFR, KDR, and SRC) were prominently enriched in the VEGF (hsa04370) and PI3K-AKT (hsa04151) pathways, with tight functional associations. This reinforced our hypothesis that PF alleviates CIRI primarily through activation of this unified endothelial regulatory axis, promoting microvascular repair and neuronal survival. Collectively, these in silico predictions provide directional clues for subsequent mechanistic validation.

Integrated network pharmacology prediction and Metascape enrichment analysis implied that PF may alleviate CIRI through multi-target regulation of VEGF and PI3K-AKT signaling. As suggested by in silico data, core mediators including EGFR, SRC, and KDR likely work together to boost angiogenesis, sustain endothelial homeostasis, and improve neuronal survival, providing directional clues for subsequent mechanistic verification.

### 2.2. PF Directly Protected SH-SY5Y Cells Against OGD/R-Induced Injury in a Concentration-Dependent Manner

**Figure 7:**
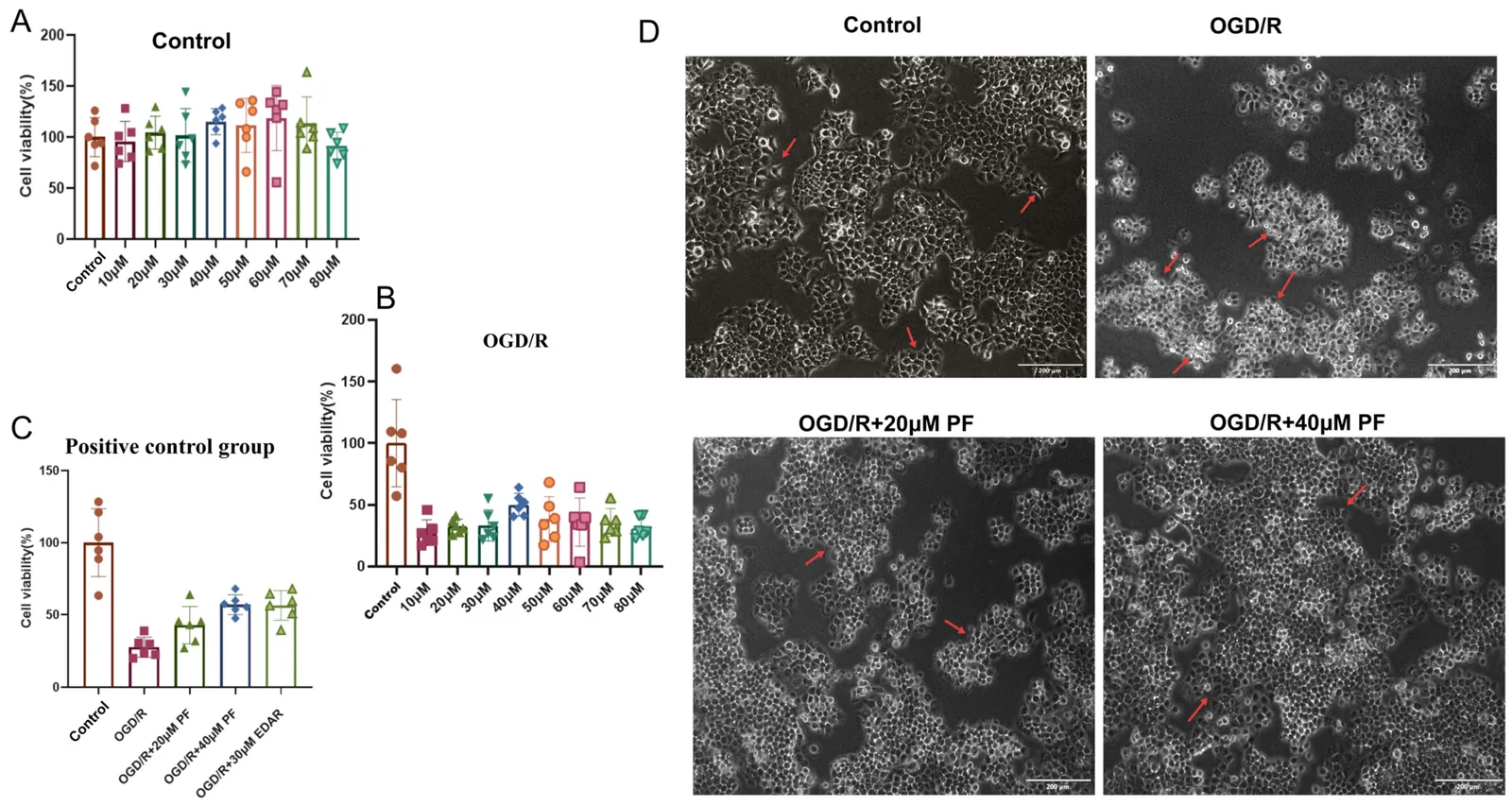
(**A**) Effect of different concentrations of PF on SH-SY5Y cell viability (MTT assay). (**B**) Effect of pretreatment with different concentrations of PF on oxygen–glucose deprivation/reoxygenation (OGD/R)-induced injury in SH-SY5Y cells (MTT assay). (**C**) Protective effect of PF on oxygen–glucose deprivation/reoxygenation (OGD/R)-induced injury in SH-SY5Y cells (MTT assay). (**D**) Representative micrographs of SH-SY5Y cells under different treatment conditions. (The regions indicated by arrows are representative of cell morphology).

The cytotoxicity of PF on SH-SY5Y cells was assessed via the MTT assay. The results illustrated that 10–80 μM PF treatment for 24 h had no significant effect on normal cell viability (Figure 7A). The OGD/R model group exhibited a decreased cell survival rate (*p* < 0.01); 40 μM PF significantly reversed this decline (Figure 7B) and ameliorated OGD/R-induced morphological damage (Figure 7D). Compared with normal cells, OGD/R-treated cells displayed reduced density, enlarged intercellular spacing, shrinkage, and rounding. In the OGD/R + 20 μM PF and OGD/R + 40 μM PF groups, cell density was increased compared with that in the OGD/R group, with attenuated shrinkage and rounding. The OGD/R + 40 μM PF group exhibited more pronounced morphological improvement, suggesting PF exerts protective effects against OGD/R-induced injury in a concentration-dependent manner.

To highlight the reliability of the experimental design and the potential for clinical translation, 30 μM edaravone [19], a clinically established neuroprotective agent, was included as a positive control (Figure 7C). The results demonstrated that 40 μM PF exerted slightly superior neuroprotective effects against OGD/R-induced neuronal injury compared with edaravone, further supporting its promising application in CIRI treatment.

### 2.3. RNA Sequencing Analysis Demonstrated That PF Mitigated OGD/R-Induced Injury Through Multi-Target Modulation of VEGF/PI3K-AKT/mTOR Signaling

**Figure 8:**
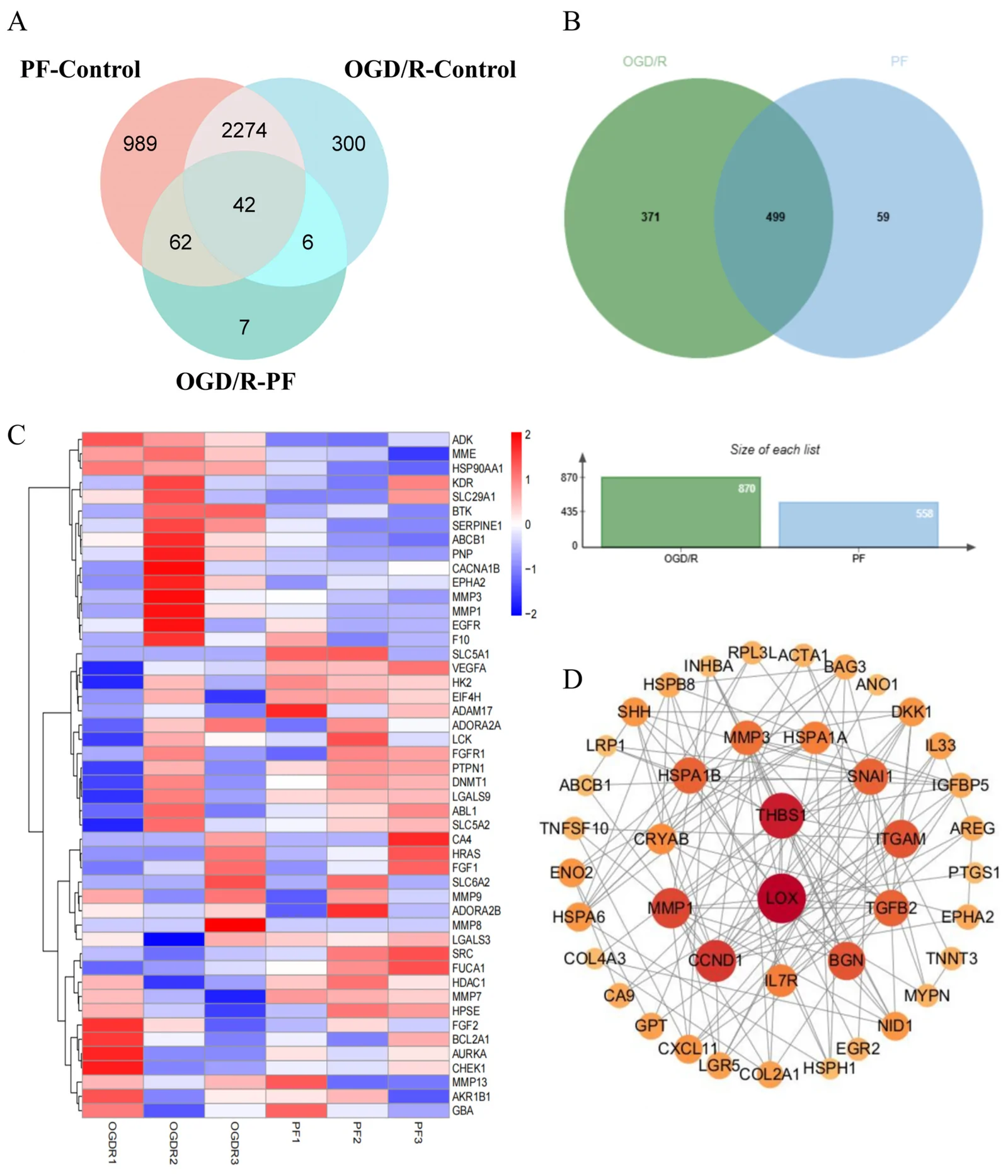
(**A**) Composite Venn diagram of differentially expressed genes (DEGs) from transcriptome sequencing of different treatment groups. (**B**) Intersection of DEGs from PF treatment and CIR model based on sequencing data. (**C**) Heatmap showing association between network pharmacology-predicted intersection targets and sequencing-derived intersection targets. (**D**) PPI network of sequencing-derived intersection targets visualized using Cytoscape.

To systematically dissect the multi-target mechanisms by which PF protects against CIRI in an OGD/R cell model, we performed RNA-seq to profile differentially expressed genes (DEGs) across all experimental groups. Venn diagrams (Figure 8A) illustrated unique and overlapping DEGs from three pairwise comparisons (PF vs. control, OGD/R vs. control, and OGD/R-PF vs. control), capturing transcripts responsive to both ischemic insult and PF intervention. OGD/R injury induced 870 DEGs relative to controls, reflecting extensive transcriptomic reprogramming; PF treatment uniquely altered 558 genes. Two-group overlap analysis identified 499 common DEGs (Figure 8B) considered candidate core functional targets because their expression was perturbed by ischemia and reversed upon PF administration.

PPI network construction and hub gene screening (Figure 8D) revealed highly connected nodes, including MMP1, MMP3, and TGFB2—known effectors in ECM degradation, BBB disruption, and VEGF-related angiogenic signaling under ischemia. CCND1 and IL7R emerged as key nodes linked to PI3K-AKT-dependent cell cycle regulation; their altered expression in PF-treated cells suggested involvement in cell cycle stabilization and apoptosis resistance. A heatmap of 44 consensus genes between transcriptomic and network pharmacology data (Figure 8C) showed coherent expression trends, with VEGF-A and SRC significantly upregulated after PF treatment, indicating reactivation of endothelial repair programs.

**Figure 9:**
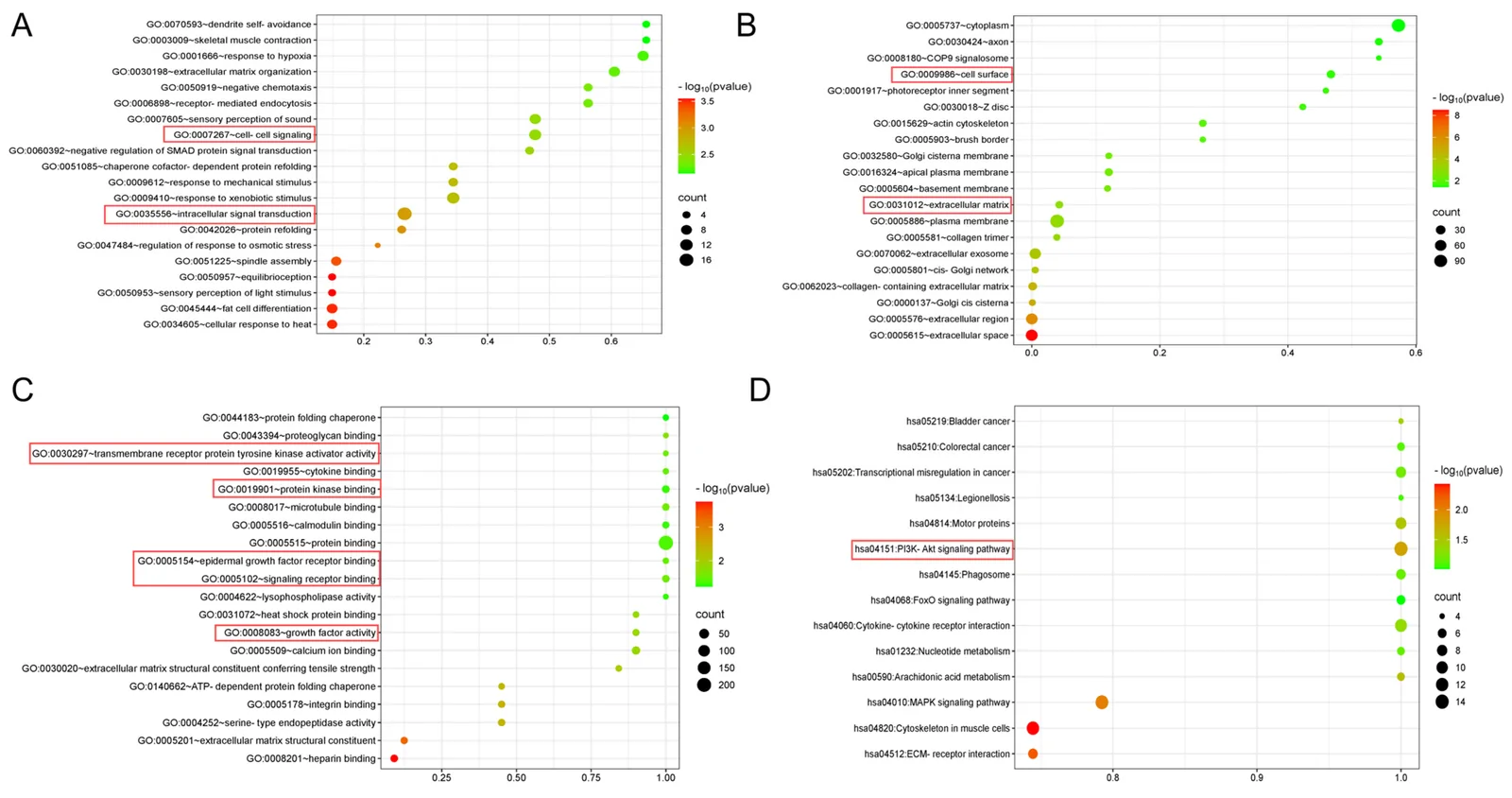
(**A**–**D**) Bubble plots of GO (BPs, CCs, MFs) and KEGG pathway enrichment analysis for the sequencing-derived intersection target genes.

**Figure 10:**
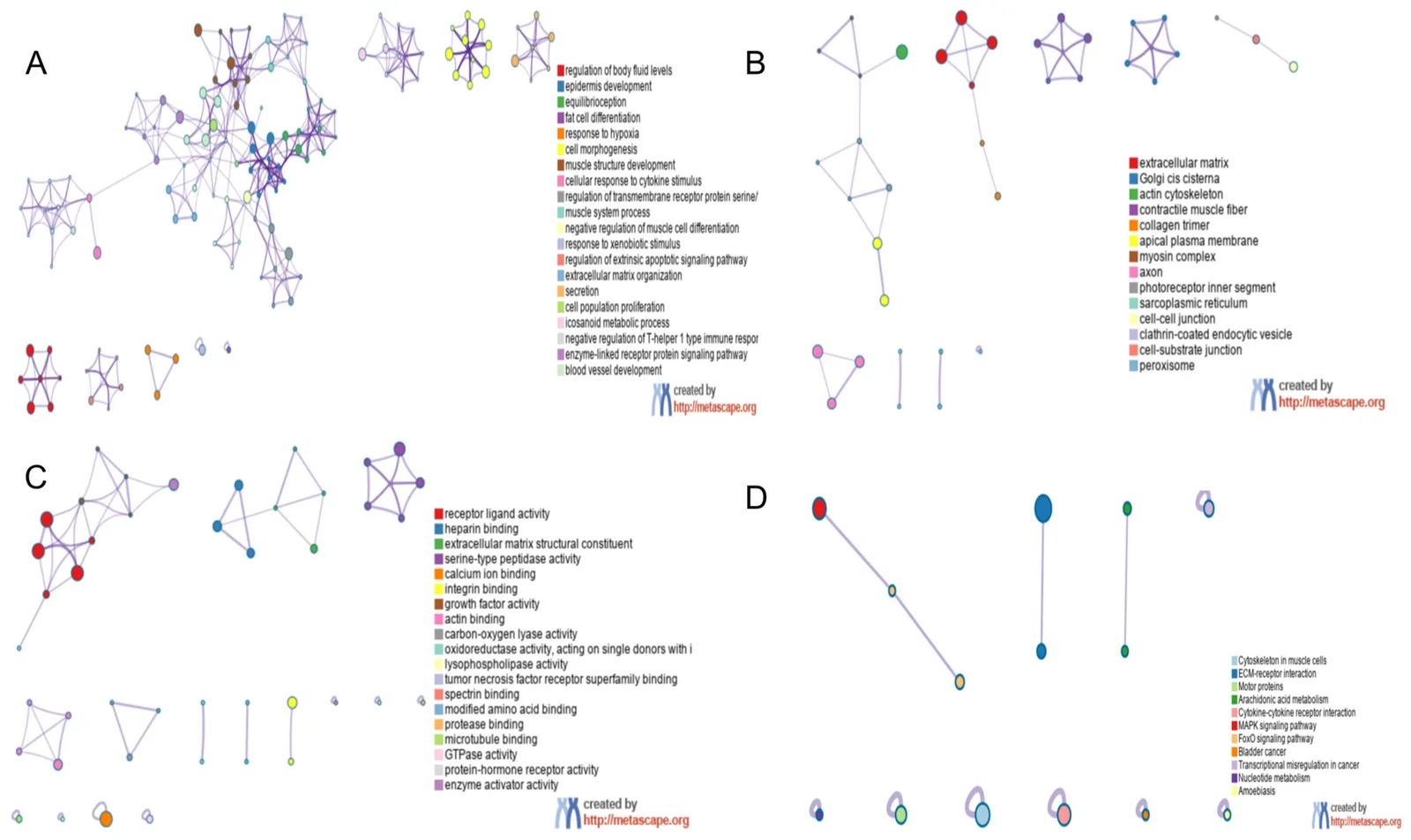
(**A**–**D**) Scatter plots of integrated GO (BPs, CCs, MFs) and KEGG enrichment results.

GO enrichment analyses (Figure 9A–C and Figure 10A–C) collectively highlighted multi-layered protective processes. Top BP terms included cell–cell signaling (GO:0007262), intracellular signal transduction (GO:0035556), blood vessel development, and cell population proliferation, consistent with VEGF-driven angiogenesis and PI3K-AKT-mediated survival. CC enrichment consistently localized PF-associated targets to the cell surface (GO:0009986), extracellular matrix (ECM; GO:0031012), and actin cytoskeleton, implicating membrane-associated and ECM-related compartments in BBB stabilization and endothelial migration. MF terms converged on growth factor binding (GO:0008083), signal receptor binding (GO:0005102), receptor ligand activity, and calcium ion binding, suggesting that PF may amplify endogenous protective signals and modulate intracellular calcium dynamics.

KEGG pathway analysis (Figure 9D and Figure 10D) pinpointed PI3K-AKT signaling (hsa04151) as a central axis, coordinating angiogenesis, metabolic homeostasis, and anti-apoptosis. Metascape-based KEGG enrichment revealed co-enrichment of mTOR and MAPK pathways alongside the VEGF/AKT axis. These three pathways form an interconnected network: VEGF/AKT primarily transduces survival signals, mTOR governs metabolic reprogramming and autophagy, and MAPK mediates adaptation to hypoxic stress. These findings indicate that PF exerts synergistic neurovascular protection through multi-pathway modulation rather than a single linear cascade.

**Figure 11:**
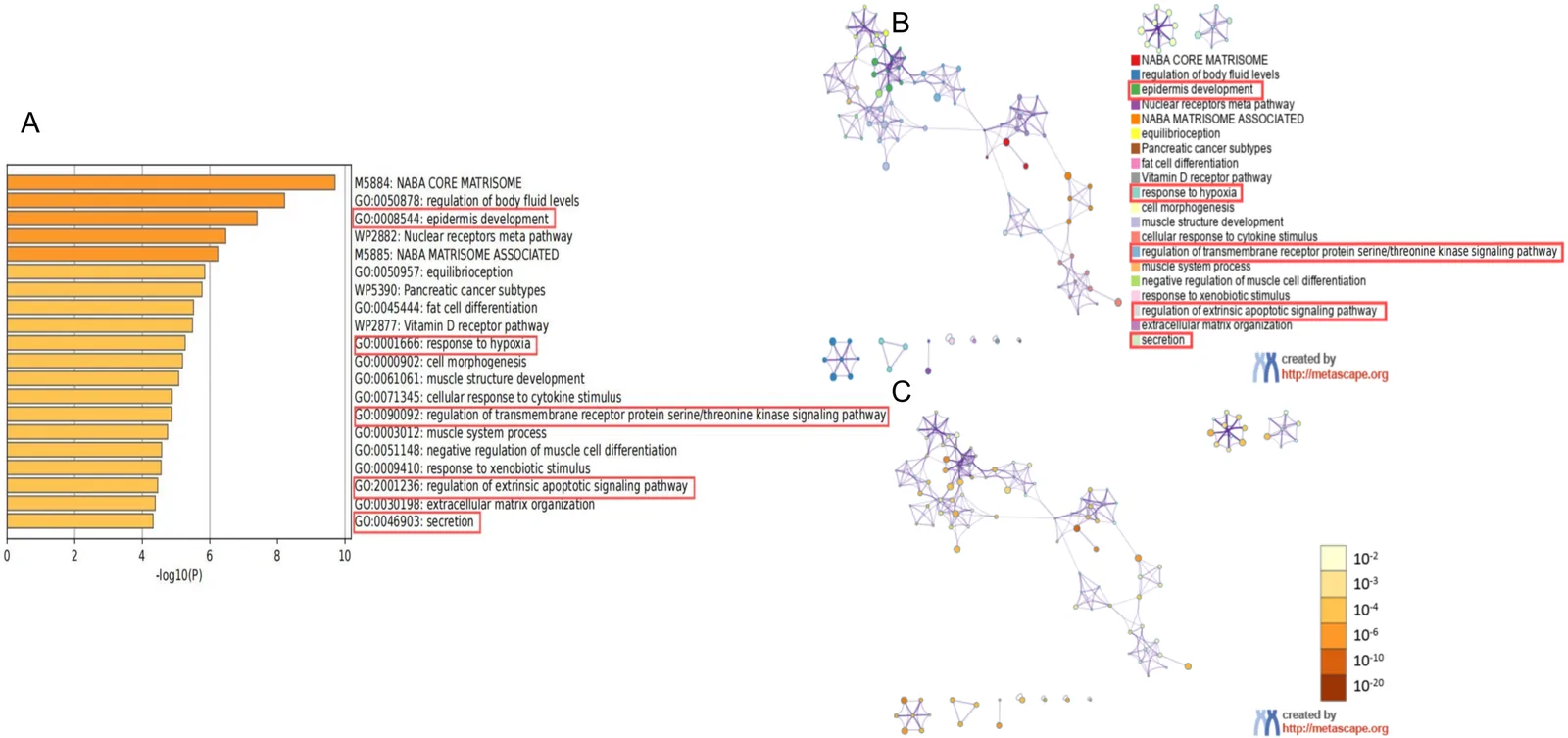
(**A**) Overview plot of enrichment analysis integrating sequencing data and meta-analysis. (**B**,**C**) Scatter plots of integrated analysis results (color intensity indicates significance level: light to dark = low to high).

Supplementary Metascape enrichment (Figure 11A–C) further associated PF targets with secretion, epidermis development, and hypoxia response. The hypoxia response directly mirrors OGD/R stress; secretion-related terms may reflect cytokine and trophic factor release that remodels the post-ischemic microenvironment, while epidermis development enrichment hints at shared transcriptional programs between epithelial and cerebrovascular endothelial cells, though its direct contribution to BBB repair warrants additional investigation.

**Figure 12:**
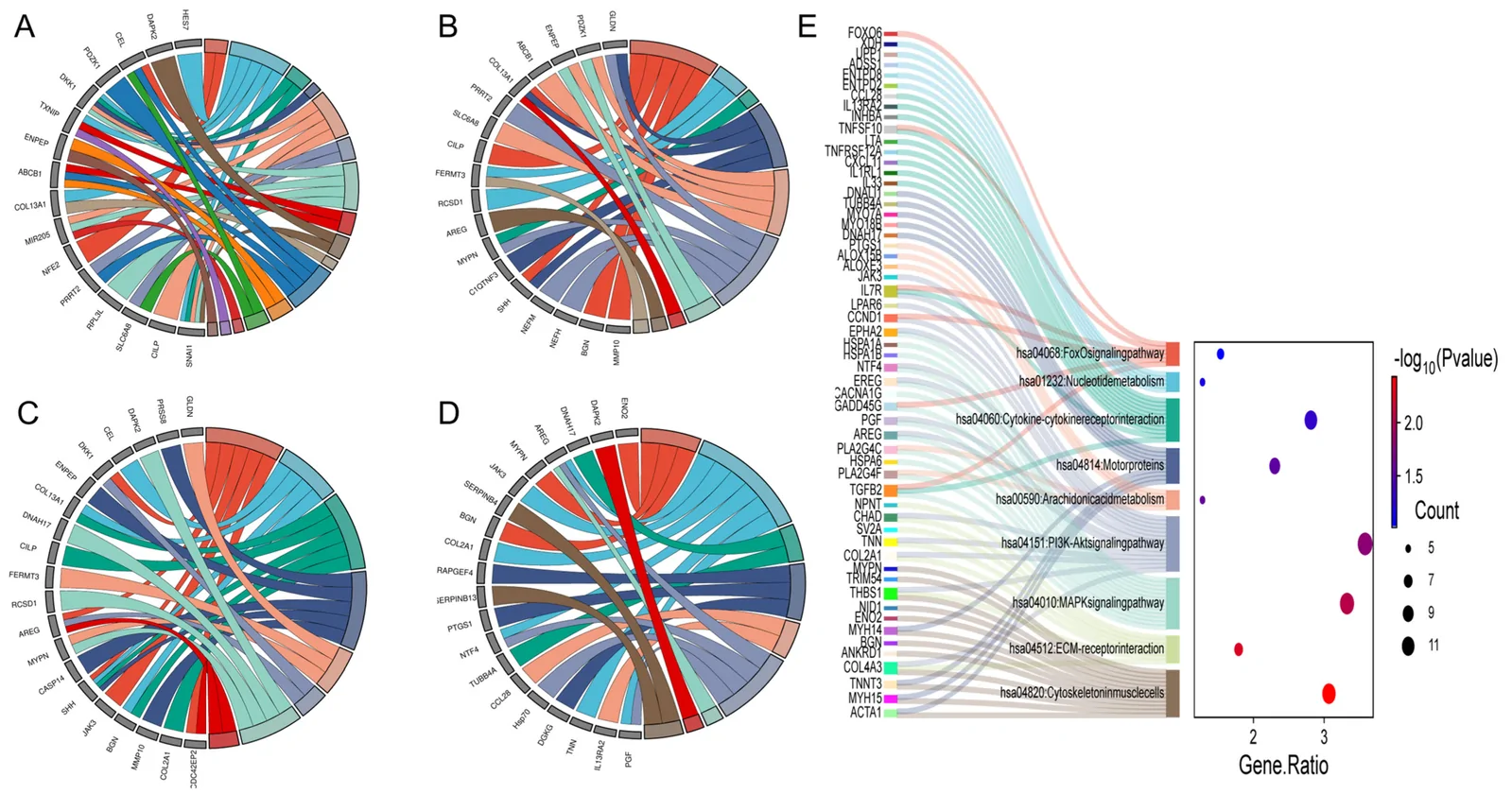
(**A**–**D**) Chord diagrams of integrated GO (BPs, CCs, MFs) and KEGG enrichment results. (**E**) Sankey bubble diagram illustrating PF–CIRI-related targets and pathways based on integrated sequencing and meta-analysis.

Chord diagrams (Figure 12A–D) and pathway–target mapping (Figure 12E) visually reinforced the strong regulatory connections between hub genes (such as COL2A1, EPHA2) and PI3K-AKT/mTOR signaling. EPHA2 is a well-characterized mediator of endothelial angiogenic activation, while COL2A1 serves as a core ECM gene shaping the perivascular matrix environment. Their tight linkage to the central pathway further substantiates the multi-target, multi-pathway mechanism of PF, improving cerebrovascular endothelial function and preserving neuronal viability to alleviate CIRI.

### 2.4. Molecular Docking Predicted High-Affinity PF Binding to the VEGF/AKT/mTOR Axis, Suggesting a Potential Role in CIRI Regulation

**Figure 13:**
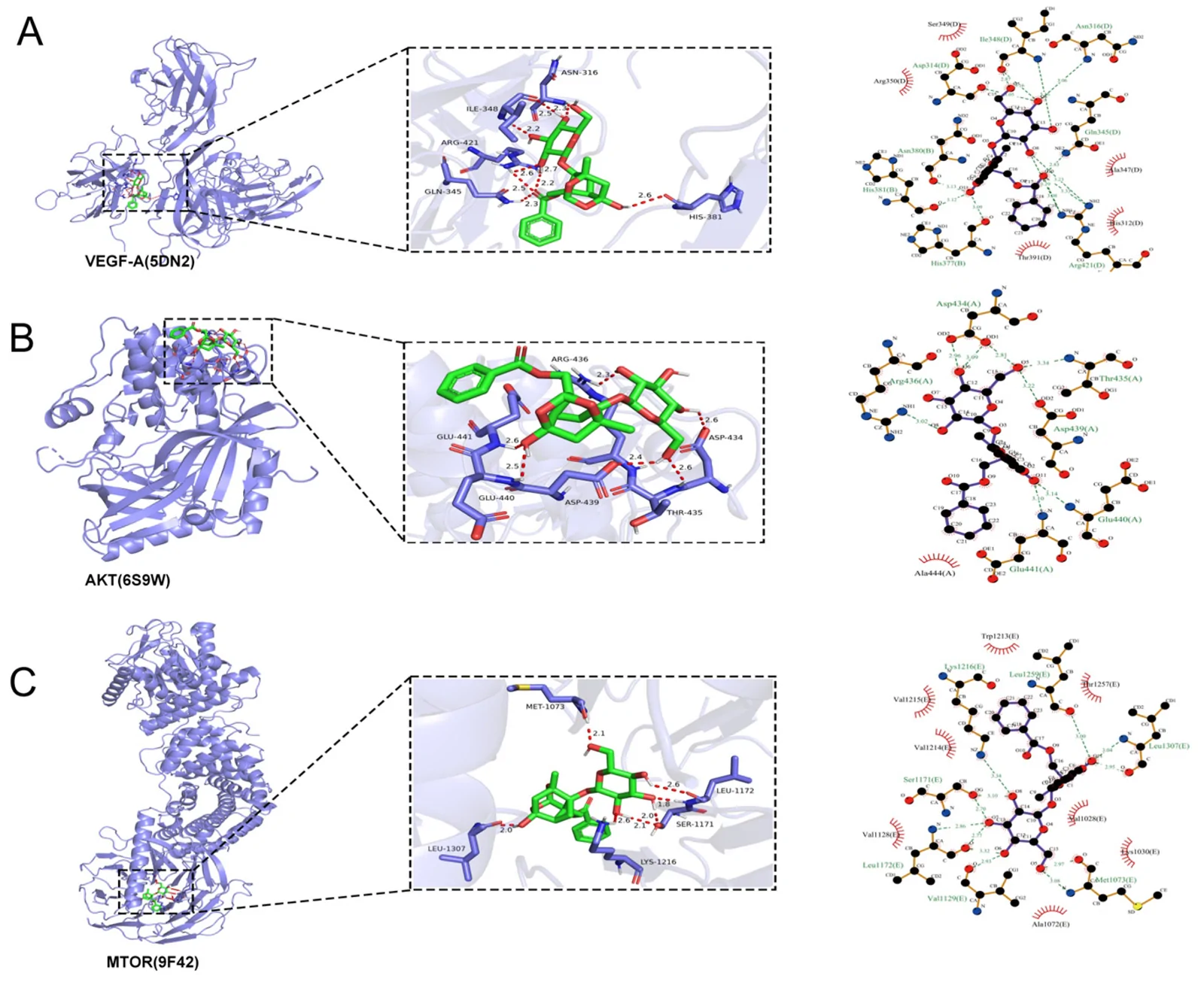
(**A**–**C**) Molecular docking modes of PF with core target proteins: VEGFA (PDB: 5DN2), AKT (PDB: 6S9W), and mTOR (PDB: 9F42). Molecular dynamics (MD) simulation analysis of PF–target protein complexes.

Molecular docking using AutoDock 4.2.6 Vina revealed several interactions. For VEGF-A (PDB: 5DN2), PF exhibited strong interactions through 13 hydrogen bonds (including Asn316 and Ile348) and six hydrophobic contacts (including Arg350), achieving a binding free energy of −8.4 kcal/mol (Figure 13A and Table 1). For AKT (PDB: 6S9W), PF formed eight hydrogen bonds (including Asp434 and Arg436) alongside one hydrophobic interaction (Ala444), yielding moderate binding affinity (−5.5 kcal/mol, Figure 13B and Table 1). The most pronounced interaction was observed with mTOR (PDB: 9F42), with which PF established 11 hydrogen bonds (most notably with Lys1216 and Leu1259) and eight hydrophobic bonds (including Trp1213 and Thr1257), resulting in an exceptionally low binding free energy of −9.6 kcal/mol (Figure 13C and Table 1). Detailed binding affinity data of PF for the target proteins VEGF-A, AKT, and mTOR are provided in Appendix A.

Based on the binding free energy threshold of <−5.0 kcal/mol for strong binding, PF showed predicted high affinity for VEGF-A and mTOR and moderate affinity for AKT. These results suggest potential CIRI regulation through VEGF/AKT/mTOR signaling.

### 2.5. Molecular Docking Indicated Predicted High-Affinity PF Binding to the VEGF/AKT/mTOR Axis for Potential Involvement in CIRI Regulation

The root mean square deviation (RMSD) is a well-established metric for evaluating protein–ligand complex conformational stability and reflects the deviation of atomic positions from their initial state. Lower RMSD values indicate higher conformational stability. Here, RMSD was employed to assess the equilibration of the simulation systems.

**Figure 14:**
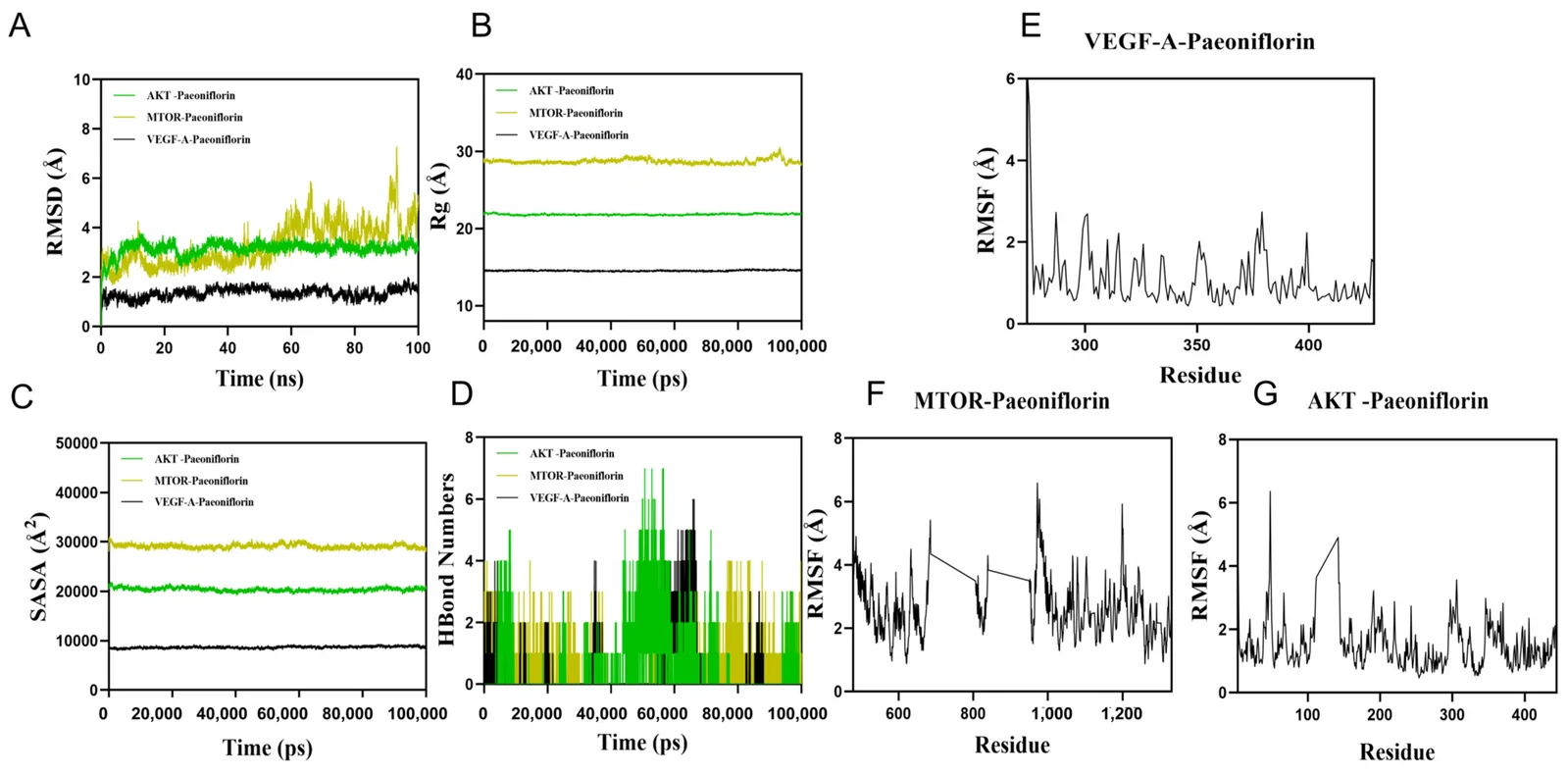
(**A**) Root mean square deviation (RMSD) of the complex backbone atoms over time. (**B**) Radius of gyration (Rg) of the complexes over time. (**C**) Solvent accessible surface area (SASA) of the complexes over time. (**D**) Number of hydrogen bonds (HBonds) within the complexes over time. (**E**–**G**) Root mean square fluctuation (RMSF) of backbone atoms for protein residues in the complexes over time.

The AKT–PF complex underwent structural relaxation during the initial 0–25 ns, with RMSD values progressively increasing as the system gradually adopted an extended conformation (Figure 14A). After 25 ns, RMSD entered a plateau of minor fluctuations, with an average value of 3.15 ± 0.27 Å throughout the stable phase, indicating that the complex reached kinetic equilibrium and maintained a stable binding conformation thereafter. The mTOR–PF complex exhibited stable RMSD fluctuations between 15 and 55 ns, suggesting a relatively stable conformation during this period; however, it displayed a slight upward trend after 55 ns, indicating minor conformational changes. The overall trajectory average RMSD was 3.28 ± 0.87 Å, with the larger standard deviation reflecting greater conformational fluctuation compared with the other two systems. In contrast, the VEGF-A–PF complex underwent only a brief structural relaxation during the first 5 ns, after which RMSD rapidly entered a stable plateau with an average value of 1.35 ± 0.20 Å and minimal oscillations. This indicates that PF binding to VEGF-A confers the strongest conformational constraint, with the fastest equilibration and the highest overall stability among the three complexes.

Further analysis revealed that the radius of gyration (Rg) and solvent-accessible surface area (SASA) of AKT–PF, mTOR–PF, and VEGF-A–PF fluctuated stably during the simulation, suggesting the absence of obvious expansion or contraction of the ligand–protein complexes throughout the motion (Figure 14B,C).

The number of hydrogen bonds formed between PF and each target protein during the simulation is presented in Figure 14D. The AKT–PF complex exhibited 0–7 hydrogen bonds, with a predominant count of approximately three bonds. The mTOR–PF complex displayed 0–4 hydrogen bonds, averaging approximately two bonds. The VEGF-A–PF complex showed 0–6 hydrogen bonds, also maintaining an average of approximately two bonds. These observations indicate favorable hydrogen-bonding interactions between PF and all three target proteins (Figure 14D).

Root mean square fluctuation (RMSF) analysis, which reflects residue flexibility, revealed relatively low RMSF values (mostly below 5 Å) for all three complexes (Figure 14E), indicating the reduced flexibility and enhanced stability of the binding interfaces.

In summary, the AKT–PF, mTOR–PF, and VEGF-A–PF complexes all exhibited stable binding. The VEGF-A–PF complex demonstrated the lowest RMSD values and maintained robust hydrogen-bonding interactions, suggesting that PF binds most favorably to the VEGF-A target protein.

### 2.6. PF Protected SH-SY5Y Cells Against OGD/R-Induced Injury Through the VEGF/PI3K-AKT/mTOR Axis

**Figure 15:**
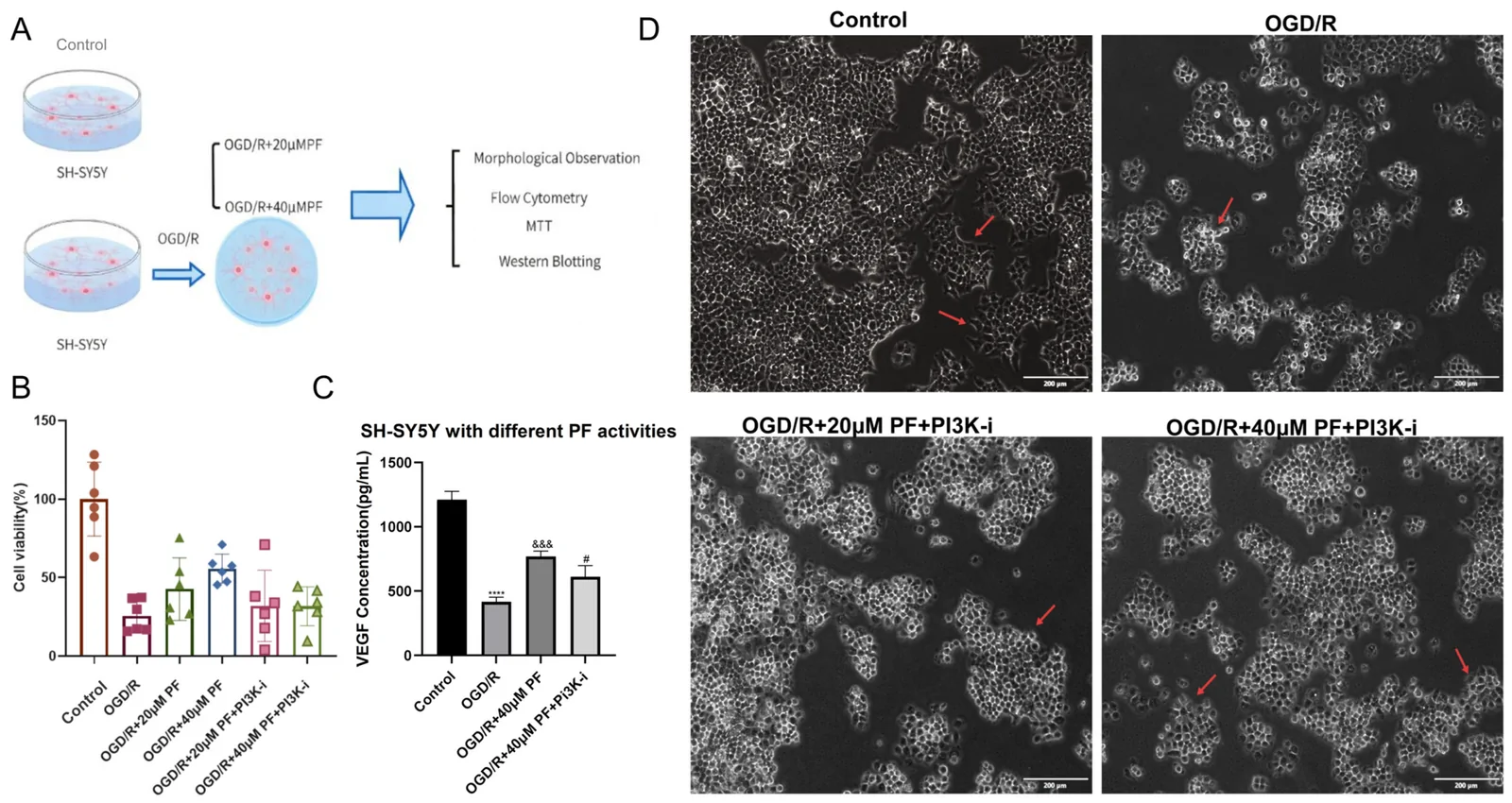
(**A**) Schematic diagram of the SH-SY5Y cell experimental protocol. (**B**) The effect of pretreatment with different concentrations of PF on OGD/R-induced SH-SY5Y cell viability (MTT assay) in the presence of inhibitors. (**C**) The levels of secreted VEGF in SH-SY5Y with different PF activities. (*n* = 3; **** *p* < 0.0001 OGD/R group vs. control group; &&& *p* < 0.001 OGD/R + 40 µM PF group vs. OGD/R group; # *p* < 0.05 OGD/R + 40 µM PF + PI3K-i group vs. OGD/R + 40 µM PF group). (**D**) Representative micrographs of SH-SY5Y cells under corresponding treatment conditions. (The regions indicated by arrows are representative of cell morphology).

**Figure 16:**
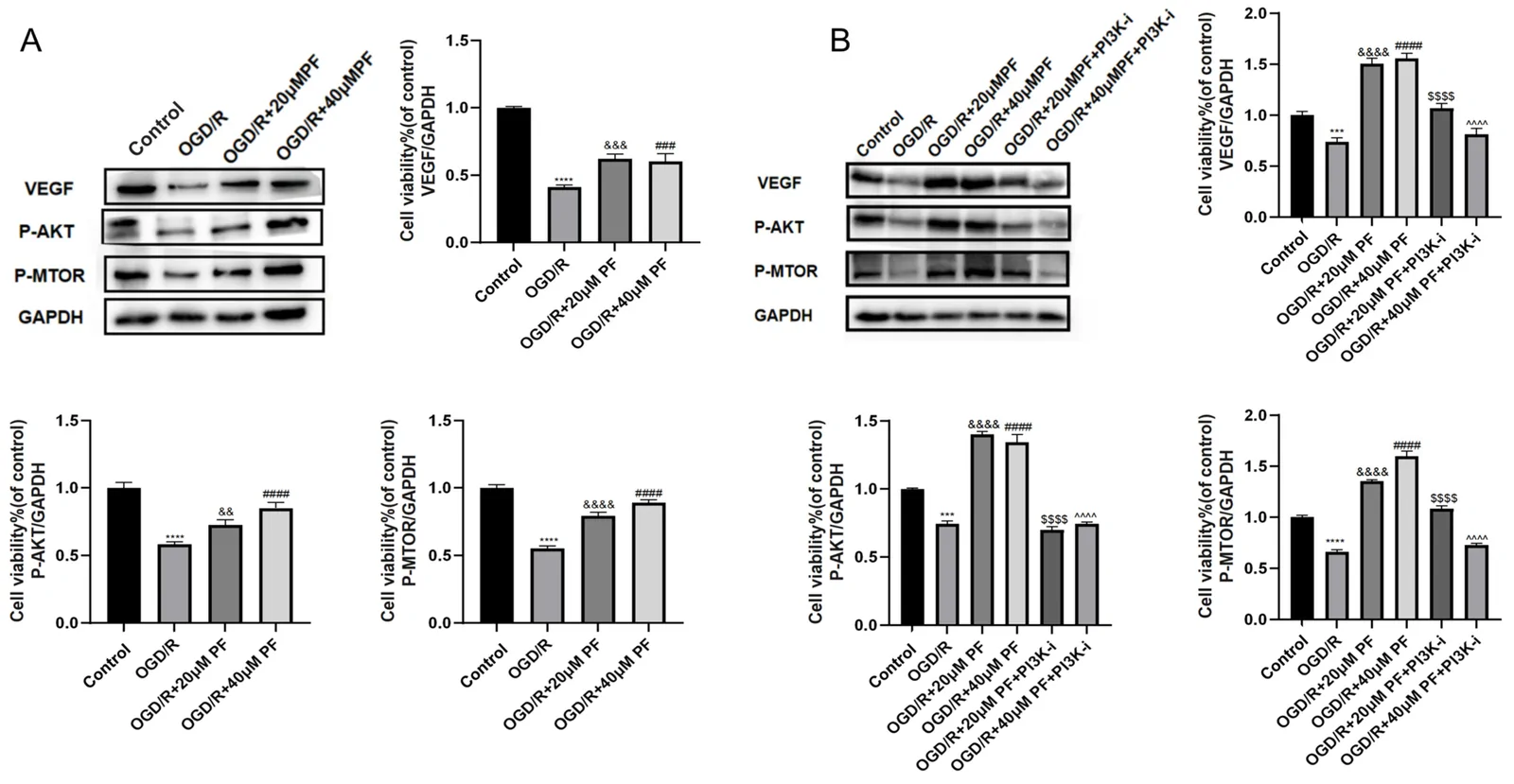
(**A**) Western blot analysis of key pathway protein expression levels and corresponding quantitative analysis (grayscale value statistics) (*n* = 3; **** *p* < 0.0001 OGD/R group vs. control group; && *p* < 0.01 OGD/R + 20 µM PF group vs. OGD/R group (for P-AKT); &&& *p* < 0.01 OGD/R + 20 µM PF group vs. OGD/R group (for VEGF); &&&& *p* < 0.0001 OGD/R + 20 µM PF group vs. OGD/R group (for P-MTOR); ### *p* < 0.001 OGD/R + 40 µM PF group vs. OGD/R group (for VEGF); #### *p* < 0.0001 OGD/R + 40 µM PF group vs. OGD/R group (for P-AKT and P-MTOR)). (**B**) Western blot analysis and quantitative analysis of key proteins under treatment with specific pathway inhibitors (*n* = 3; *** *p* < 0.001 OGD/R group vs. control group; **** *p* < 0.0001 OGD/R group vs. control group; &&&& *p* < 0.0001 OGD/R + 20 µM PF group vs. OGD/R group; #### *p* < 0.0001 OGD/R + 40 µM PF group vs. OGD/R group; $$$$ *p* < 0.0001 OGD/R + 20 µM PF + PI3K-i group vs. OGD/R + 20 µM PF group; ^^^^ *p* < 0.0001 OGD/R + 40 µM PF + PI3K-i group vs. OGD/R + 40 µM PF group).

SH-SY5Y cells were treated according to the experimental protocol shown in Figure 15A. Western blotting (Figure 16A) demonstrated that VEGF, p-AKT, and p-mTOR protein expression significantly decreased in the OGD/R model group (*p* < 0.01 vs. control). PF increased the expression of these proteins in a concentration-dependent manner (*p* < 0.01 vs. the OGD/R group), indicating that it exerts its effects by activating VEGF/PI3K-AKT/mTOR signaling.

Compared with the findings in the PF monotherapy groups (20 and 40 μmol·L^−1^, Figure 16B), co-administration of the PI3K inhibitor LY294002 significantly reversed PF-induced upregulation of VEGF, AKT, and mTOR (all *p* < 0.05). In parallel with the Western blotting results, VEGF levels in the supernatant of SH-SY5Y cells under different PF treatments were detected by enzyme-linked immunosorbent assay (ELISA). OGD/R treatment reduced VEGF release, whereas PF administration increased it (Figure 15C). Cell viability was reduced in the OGD/R model group (*p* < 0.01 vs. control, set as 100%). PF treatment (20 and 40 μmol·L^−1^) alleviated the reduction in cell viability induced by OGD/R in a concentration-dependent manner (*p* < 0.01 vs. the OGD/R group). However, its protective effect was largely abolished by LY294002 (*p* < 0.05 vs. PF alone), confirming the pivotal role of the PI3K pathway.

**Figure 17:**
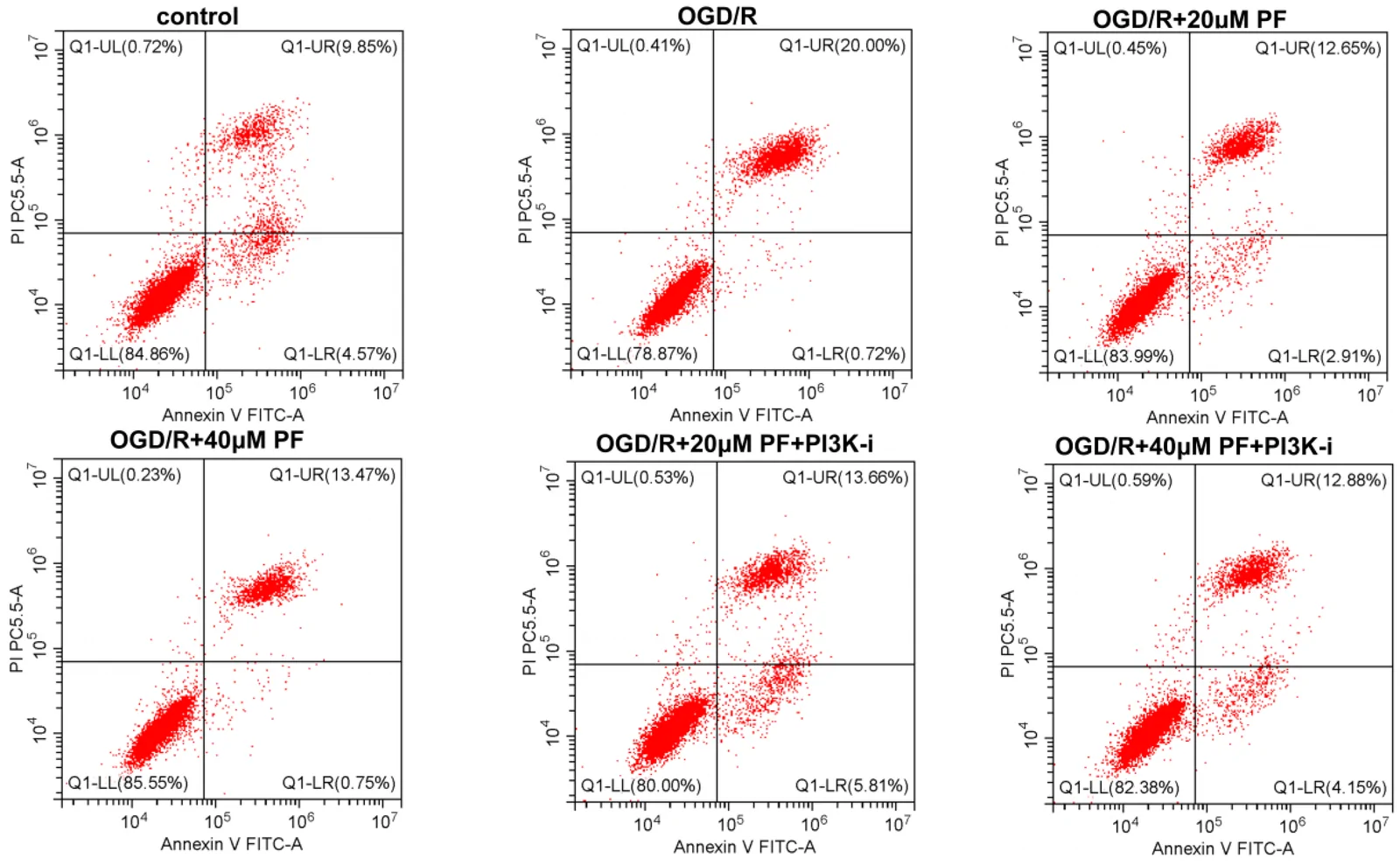
Flow cytometry analysis of apoptosis in OGD/R-induced SH-SY5Y cells pretreated with PF in the presence of inhibitors.

Morphological analysis (Figure 15D) further supported these findings. OGD/R-exposed cells exhibited characteristic damage (including shrinkage and synapse loss), whereas PF-treated cells displayed preserved morphology; this improvement was diminished upon PI3K inhibition. Flow cytometry revealed a significant increase in apoptosis in the OGD/R group (*p* < 0.01 vs. control), which was reduced by approximately 57% by 40 μmol·L−1 PF (*p* < 0.01 vs. OGD/R group, Figure 17). LY294002 partially counteracted PF’s anti-apoptotic effects (*p* < 0.05 vs. PF alone, Figure 15B). These results demonstrate that PF protects SH-SY5Y cells from OGD/R-induced injury by activating VEGF/PI3K-AKT/mTOR signaling.

### 2.7. PF Alleviates OGD/R-Induced Damage in HCMEC/D3 Cells Through VEGF/PI3K-AKT/mTOR Signaling

**Figure 18:**
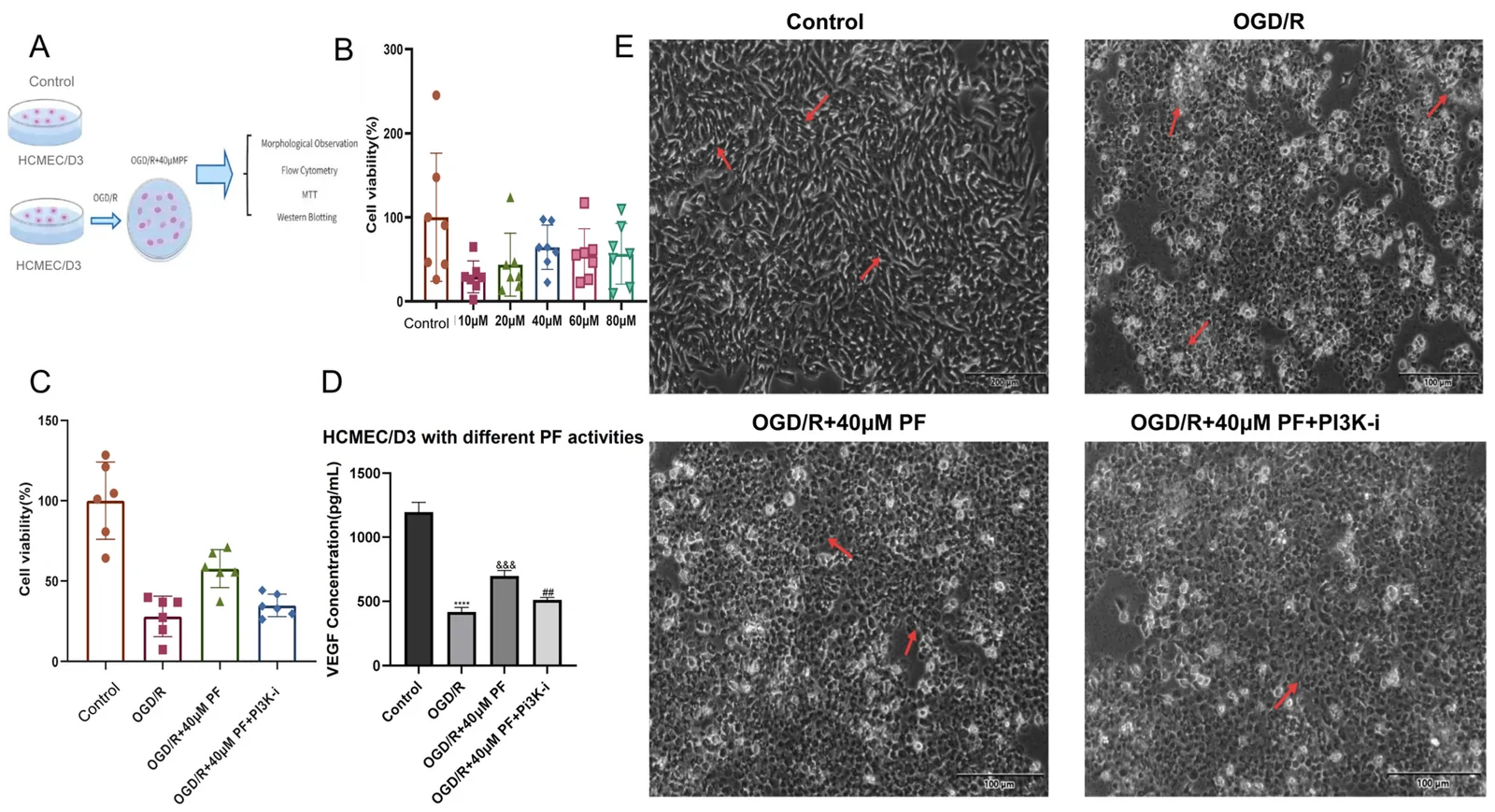
(**A**) Schematic diagram of the HCMEC/D3 cell experimental protocol. (**B**) The effect of pretreatment with different concentrations of PF on OGD/R-induced HCMEC/D3 cell viability (MTT assay) (**C**) The effect of pretreatment with different concentrations of PF on OGD/R-induced HCMEC/D3 cell viability (MTT assay) in the presence of inhibitors. (**D**) The levels of secreted VEGF in SH-SY5Y with different HCMEC/D3 CM activities. (*n* = 3; **** *p* < 0.0001 OGD/R group vs. control group; &&& *p* < 0.001 OGD/R + 40 µM PF group vs. OGD/R group; ## *p* < 0.01 OGD/R + 40 µM PF + PI3K-i group vs. OGD/R + 40 µM PF group). (**E**) Representative micrographs of HCMEC/D3 cells under corresponding treatment conditions. (The regions indicated by arrows are representative of cell morphology).

**Figure 19:**
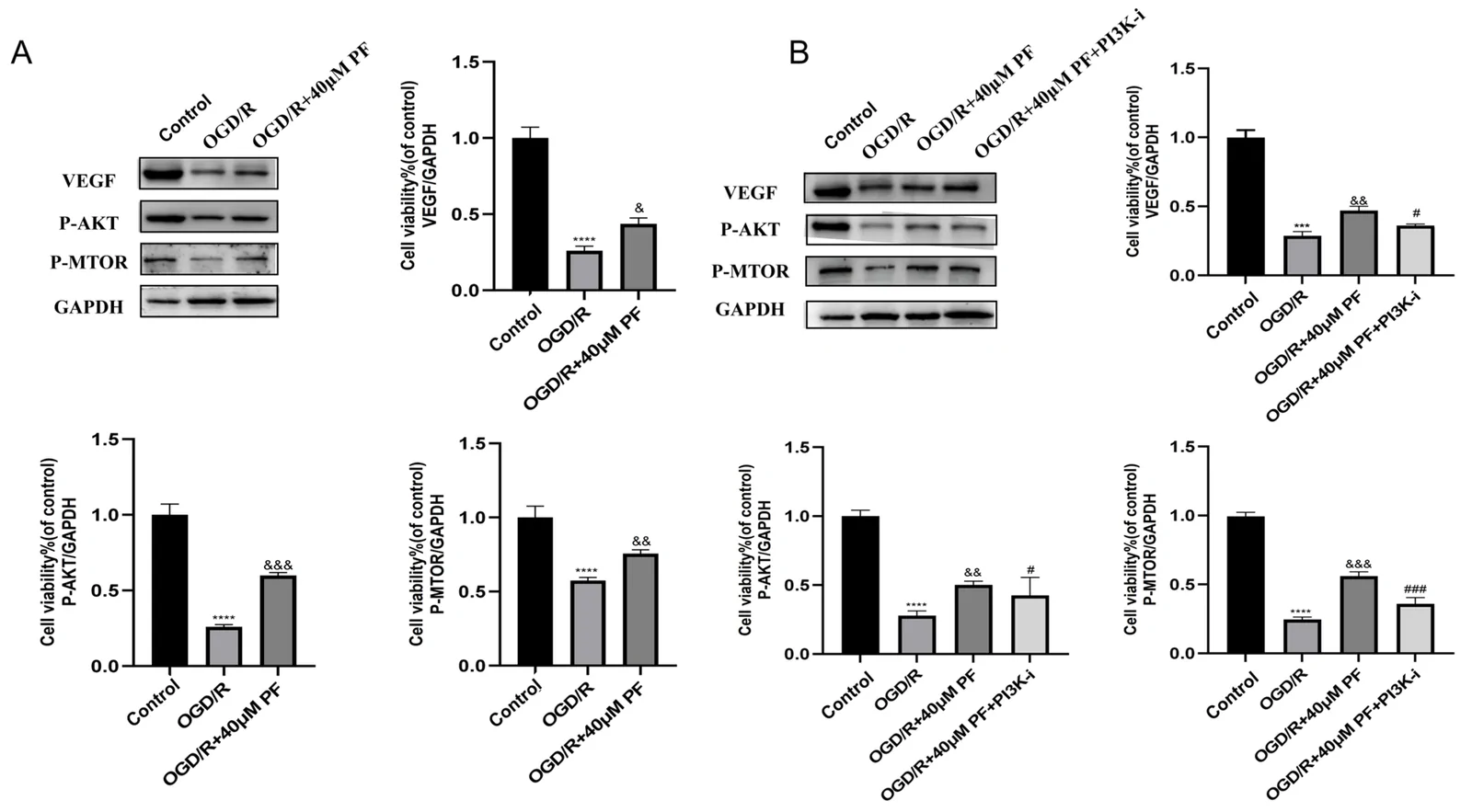
(**A**) Western blot analysis of key pathway protein expression levels and corresponding quantitative analysis (grayscale value statistics) (*n* = 3; **** *p* < 0.0001 OGD/R group vs. control group; & *p* < 0.05 OGD/R + 40 µM PF group vs. OGD/R group (for VEGF); && *p* < 0.01 OGD/R + 40 µM PF group vs. OGD/R group (for P-MTOR); &&& *p* < 0.001 OGD/R + 40 µM PF group vs. OGD/R group (for P-AKT)). (**B**) Western blot analysis and quantitative analysis of key proteins under treatment with specific pathway inhibitors. (*n* = 3; *** *p* < 0.001 OGD/R group vs. control group (for VEGF); **** *p* < 0.0001 OGD/R group vs. control group (for P-AKT); && *p* < 0.01 OGD/R + 40 µM PF group vs. OGD/R group (for VEGF and P-AKT); &&& *p* < 0.001 OGD/R + 40 µM PF group vs. OGD/R group (for P-MTOR); # *p* < 0.05 OGD/R + 40 µM PF + PI3K-I group vs. OGD/R + 40 µM PF group (for VEGF and P-AKT); ### *p* < 0.001 OGD/R + 40 µM PF + PI3K-igroup vs. OGD/R + 40 µM PF group (for P-MTOR)).

Following the experimental protocol depicted in Figure 18A, HCMEC/D3 cells were subjected to OGD/R injury and treated with PF. MTT assays (Figure 18B) revealed that OGD/R significantly reduced cell viability (*p* < 0.01 vs. control), which was ameliorated by 10–80 μmol·L^−1^ PF in a concentration-dependent manner (*p* < 0.01 vs. OGD/R). Morphological observation (Figure 18E) confirmed that OGD/R induced cell shrinkage and synapse loss, whereas 40 μmol·L^−1^ PF preserved cellular integrity—an effect abolished by co-administration of the PI3K inhibitor LY294002. Western blotting (Figure 19A,B) demonstrated that OGD/R downregulated VEGF, P-AKT, and P-mTOR expression (*p* < 0.01 vs. control), while PF upregulated them (*p* < 0.01 vs. OGD/R); LY294002 significantly reversed PF-induced protein upregulation and viability restoration (all *p* < 0.05 vs. PF alone).

**Figure 20:**
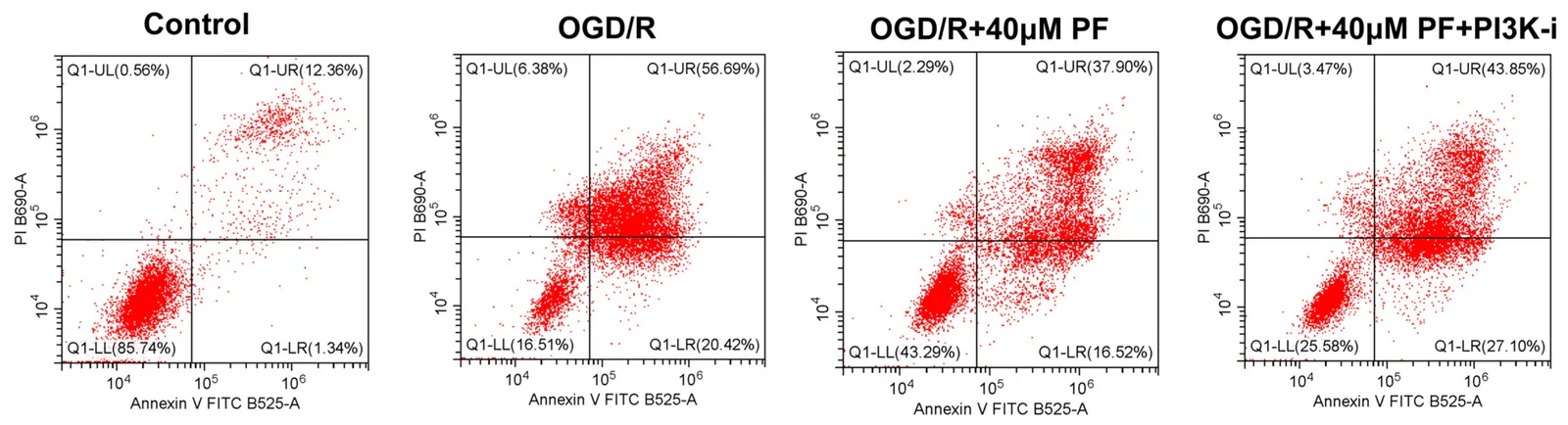
Flow cytometry analysis of apoptosis in OGD/R-induced HCMEC/D3 cells pretreated with PF in the presence of inhibitors.

Complementing these findings, VEGF levels in the supernatant of HCMEC/D3 cells under different PF treatments were detected by ELISA. OGD/R treatment reduced VEGF release, whereas PF administration increased it (Figure 18D). Flow cytometry (Figure 20) showed that OGD/R increased apoptosis (*p* < 0.01 vs. control), while 40 μmol·L^−1^ PF reduced apoptosis by 33% (*p* < 0.01 vs. OGD/R), an effect partially counteracted by LY294002 (*p* < 0.05 vs. PF alone). These findings indicate that PF protected HCMEC/D3 cells against OGD/R injury through VEGF/PI3K-AKT/mTOR signaling activation.

### 2.8. PF Alleviates CIRI by Mediating Crosstalk Between Neurons and Endothelial Cells Through the VEGF/PI3K-AKT/mTOR Pathway

**Figure 21:**
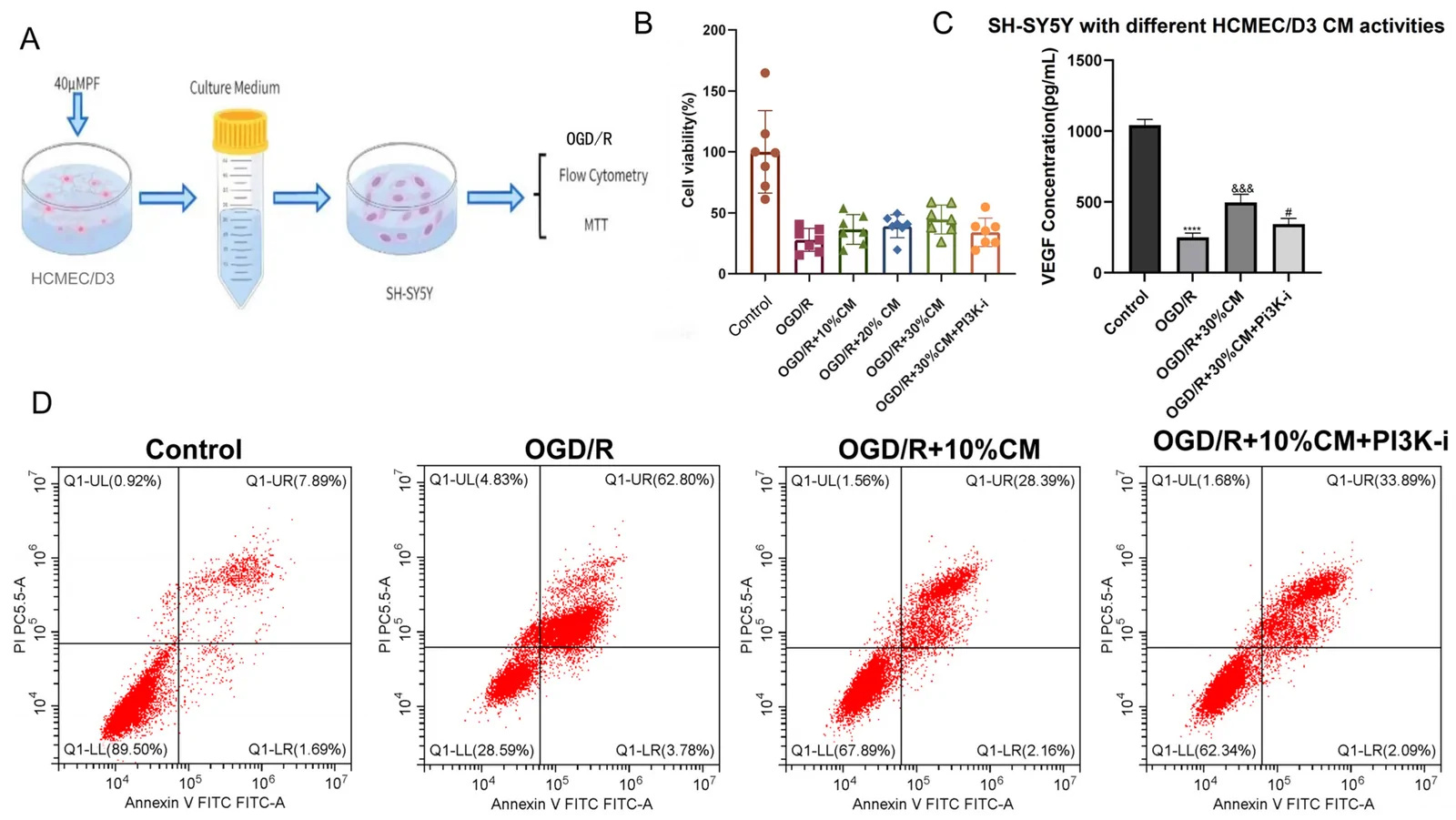
(**A**) Schematic of the protocol used for observing the crosstalk between endothelial cells and neurons regulated by PF. (**B**) Effect of different concentrations of the culture supernatant of HCMEC/D3 cells on OGD/R-induced SH-SY5Y cell viability (MTT assay). (**C**) Levels of secreted VEGF in SH-SY5Y cells with different HCMEC/D3 CM activities. (*n* = 3; **** *p* < 0.0001 OGD/R group vs. control group; &&& *p* < 0.0001 OGD/R + 30% CM group vs. OGD/R group; # *p* < 0.05 OGD/R + 30% CM + PI3K-I group vs. OGD/R + 30% CM group) (**D**) Flow cytometry analysis of apoptosis in OGD/R-induced SH-SY5Y cells treated with HCMEC/D3 cell culture supernatant.

The preceding experiments suggested the direct effects of PF on neuronal and endothelial cells. Network pharmacology and related analyses further revealed the potential influence of the vascular endothelial cell microenvironment on neuronal protection. To investigate this, we built a co-culture model with HCMEC/D3 and SH-SY5Y to investigate the impact of HCMEC/D3 on neuronal SH-SY5Y (Figure 21A). After HCMEC/D3 cells were exposed to 40 μM PF, the conditioned medium was collected and prepared at various concentrations. MTT assays were then performed to determine the optimal concentration of conditioned medium for subsequent experiments. Under 10% CM, cell viability was elevated compared with that in the OGD/R group (*p* < 0.05) (Figure 21B). Conditioned medium from PF-treated HCMEC/D3 cells increased SH-SY5Y cell viability. Additionally, VEGF levels in the supernatant of SH-SY5Y cells treated with different conditioned media were detected by ELISA. OGD/R treatment reduced VEGF release, whereas PF administration increased it (Figure 21C). This protective effect was partially abolished upon addition of the Pi3K inhibitor (Figure 21D).

## 3. Discussion

CIRI, a critical complication following ischemic stroke, remains a major clinical challenge because of its intricate pathophysiological mechanisms and limited response to treatment [21]. Triggered by reperfusion-induced tissue damage, CIRI involves oxidative stress, BBB disruption, and neuroinflammation. Current interventions are constrained by narrow therapeutic windows, systemic side effects, and single-target mechanisms [22,23], and nearly 40% of patients suffer secondary neurological deterioration. Novel multi-target therapies are urgently needed to address core processes such as survival signaling, angiogenesis, and ECM repair.

PF, a monoterpenoid glycoside isolated from *P. lactiflora*, has emerged as a promising neuroprotective agent with demonstrated anti-inflammatory, antioxidant, and anti-apoptotic properties [24,25,26,27]. PF exhibited neuroprotection in rat models of cerebral ischemia through impacting the cytochrome c/caspase 3/HDAC4 pathway [28]. Additionally, PF activates autophagy through MAPK/mTOR signaling to suppress oxidative stress and apoptosis [29,30], and was found to alleviate apoptosis and inflammation in cisplatin-induced acute kidney injury by promoting a PPI network between Hsp90AA1 and AKT [31]. However, these PF studies primarily focused on isolated pathways, leaving systemic mechanisms and crosstalk between angiogenesis and survival pathways underexplored.

This study employed an integrated strategy combining network pharmacology, molecular dynamics, and cellular experiments to systematically elucidate the multi-target mechanisms of PF in CIRI. Network pharmacology identified 44 shared targets between PF and CIRI, with the VEGF/PI3K-AKT/mTOR axis serving as a central hub. This finding aligns with previous reports of PF attenuating neuronal apoptosis through PI3K-AKT activation, and further identifies VEGF-driven angiogenesis as a synergistic mechanism. Transcriptomic analysis demonstrated PF-mediated regulation of ECM remodeling genes (such as MMP1 and TGFB2) and pro-angiogenic factors (including VEGFA), suggesting its dual role in vascular repair and barrier stabilization, a dimension overlooked in earlier studies.

Although earlier studies elucidated the neuroprotective effects of PF through isolated pathways (including CaMKII/CREB or HDAC4), the multiomics approach used here revealed a broader regulatory network. We further demonstrate for the first time that PF can directly act on BBB endothelial cells, a population critical for maintaining neurovascular integrity. Transcriptomic sequencing identified 499 PF-modulated DEGs, including those involved in ECM degradation (MMP1) and cell cycle regulation (CCND1). This extends the known functions of PF to ECM homeostasis, a critical process for preventing hemorrhagic transformation during reperfusion. GO and KEGG analyses further indicated enrichment of PF in hypoxia response pathways (including HIF1 and NF-κB) and growth factor signaling, suggesting its potential to amplify endogenous repair mechanisms through positive feedback loops. This is the first study to systematically delineate the molecular network of PF involving the VEGF/PI3K-AKT/mTOR axis. These results resonate with recent advances highlighting the role of PI3K-AKT signaling in regulating cellular survival and metabolic homeostasis in ischemic stroke [32]. The observed ECM remodeling might reflect the dual capacity of PF to mitigate BBB disruption through VEGF-driven angiogenesis while modulating MMP activity, a critical balance for preventing hemorrhagic transformation during reperfusion [33]. This study transcends the single-target paradigm by demonstrating simultaneous regulation of VEGF/PI3K-AKT/mTOR signaling by PF. This dual activation might explain the superiority of PF over single-target inhibitors (including mTOR inhibitors such as rapamycin) in suppressing OGD/R-induced apoptosis; balanced mTOR and VEGF activation could promote angiogenesis while maintaining autophagic flux, a synergistic effect vital for long-term functional recovery.

Beyond direct pathway activation, the therapeutic efficacy of PF in CIRI also derives from its ability to modulate the neurovascular microenvironment. The neurovascular unit, composed of neurons, endothelial cells, and the ECM, undergoes homeostatic disruption that exacerbates secondary injury following ischemia–reperfusion [34,35]. Transcriptomic analysis in this study revealed that PF regulated multiple genes involved in ECM remodeling (including MMP1, MMP3, and TGFB2) and endothelial function (including VEGFA, SRC, and KDR), suggesting that PF exerts a coordinated regulatory effect on neurovascular unit homeostasis. Uncontrolled ECM degradation compromises BBB integrity, while appropriate ECM remodeling is essential for angiogenesis and tissue repair [36]. The dual regulation of matrix metalloproteinases and pro-angiogenic factors by PF may represent a finely tuned mechanism balancing vascular permeability with reparative angiogenesis.

Co-culture experiments demonstrated that conditioned medium from PF-treated HCMEC/D3 cells significantly enhanced the viability of OGD/R-injured SH-SY5Y neurons, an effect that was partially reversed by a PI3K inhibitor, indicating that PF-induced endothelial activation exerts neuroprotective effects through the PI3K-AKT pathway. Collectively, these findings suggest that PF not only directly activates neuronal survival pathways but also indirectly promotes neuroprotection by remodeling the endothelial microenvironment, a dual mechanism that may confer unique therapeutic advantages in the context of multicellular dysfunction characteristic of CIRI. The translational potential of PF lies in its low cytotoxicity and multi-target regulatory capacity. By concurrently enhancing survival signaling (PI3K-AKT) and inhibiting apoptotic drivers (such as caspase 3), PF addresses the multifactorial pathology of CIRI, offering therapeutic advantages over conventional agents.

This study had several limitations warranting attention. Because SH-SY5Y and HCMEC/D3 cells were used, functional recovery endpoints and sex-stratified analyses in animal models are required to confirm our in vitro findings. Additionally, interactions between PF and Wnt/Notch pathways (which intersect with VEGF in neurovascular remodeling) remain unexplored; clarifying such crosstalk could further enrich our understanding of the mechanism of PF in brain-related pathologies. In addition, LY294002 was used to inhibit PI3K activity in cell experiments and Western blot assays. As an ATP-competitive inhibitor, LY294002 exhibits limited specificity and can also inhibit multiple kinases including CK2, mTOR, and DNA-PK; therefore, the observed results may be partially attributable to off-target effects beyond PI3K. This study also did not include VEGF-specific inhibition or rescue experiments (such as using anti-VEGF antibody, VEGFR2 inhibitor, or VEGF knockdown). Because of this, whether VEGF is an obligatory upstream regulator of the PF-mediated PI3K-Akt/mTOR cascade or merely a responsive factor among multiple targets remains to be established. Lastly, this study used a conditioned medium assay to evaluate the neuroprotective effects of PF; however, residual PF molecules may have been transferred with the medium, producing a carry-over effect. Because of this, the current design cannot distinguish between direct PF action and indirect paracrine-mediated effects, limiting the conclusion that PF indirectly modulates intercellular crosstalk.

This study provided a framework for integrating traditional medicine with systems biology, emphasizing the need for precision medicine approaches in herbal compound research. Future work should prioritize in vivo pharmacokinetics and neuroimaging to assess the brain permeability, distribution characteristics, and accumulation of PF, clarifying central nervous system exposure. Subsequently, in vivo validation assessing its effects on cognitive deficits in rodent stroke models and motor recovery should be performed. Additionally, histopathology and immunofluorescence should be used to elucidate neuroprotective mechanisms, along with omics expansion elucidating off-target effects and metabolic regulation through proteomics and metabolomics. Dedicated studies using in vitro BBB models and in vivo pharmacokinetics should be performed. Future studies should use Transwell co-cultures to exclude residual PF and validate the paracrine mechanism, or dialyze conditioned medium before functional assays. Additionally, evaluations of synergistic efficacy by co-administering PF with recombinant tissue plasminogen activator (rtPA) or antiplatelet agents should be conducted to assess combined neuroprotective efficacy and clarify the protective effects of combination therapy on post-stroke neurological function as well as the underlying synergistic mechanisms, extending therapeutic windows.

## 4. Materials and Methods

**Chemicals and reagents**

PF (CAS: 23180-57-6; C_23_H_28_O_11_; MW 480.46; purity ≥ 98%; MedChemExpress, Shanghai, China) and LY294002 (PI3K inhibitor; CAS: 154447-36-6; C_19_H_19_NO_3_; MW 307.36; purity ≥ 98%; Beyotime, Shanghai, China) were dissolved in DMSO.

**Cell culture**

Cells were obtained from the Shanghai Institute of Cell Biology, Chinese Academy of Sciences (Shanghai, China). Cells were cultured in DMEM supplemented with 10% fetal bovine serum, 2 mmol·L^−1^/L glutamine (Gibco, Grand Island, NY, USA), penicillin (100 U·mL^−1^), and streptomycin (100 μg/mL), and maintained at 37 °C and 5% CO_2_ in a humid environment. The medium was replaced twice each week.

**Western blotting**

Cells cultured in six-well cell culture plates were initially rinsed thoroughly with phosphate-buffered saline (1 × PBS, pH 7.4), and whole-cell lysates were obtained using RIPA lysis buffer (Solarbio, Beijing, China). After centrifugation (10,000 r for 4 min), the supernatants containing the protein lysates were collected. Equal amounts of protein from the whole-cell lysates were first resolved by sodium dodecyl sulfate–polyacrylamide gel electrophoresis and then transferred onto polyvinylidene difluoride membranes. The membranes were blocked for 1 h in milk (0.1 g·mL^−1^) and subsequently incubated with the primary antibody (from Beyotime) at 4 °C overnight. Membranes were then washed three times with Tris-buffered saline–Tween and incubated with the anti-rabbit secondary antibody (from Proteintech, Wuhan, China) for 2 h. The signal was detected using an electrochemiluminescence detection reagent (Solarbio).

**Flow cytometry**

For each experimental group, supernatant was collected from the cell culture medium and transferred to a 5 mL centrifuge tube. The cells were thoroughly rinsed with PBS and detached using trypsinization. After digestion, 2 mL of trypsin was neutralized, and the cell suspension was transferred to a fresh centrifuge tube. The cell suspension was centrifuged, the supernatant was discarded, and the cells were washed with PBS and centrifuged again. The cell pellet was resuspended in 1 mL of cell staining buffer; 100 µL of resuspended cells were aliquoted and mixed with 5 µL of Annexin V, and the mixture was incubated at 25 °C temperature for 10 min. Subsequently, 5 µL of propidium iodide (PI) staining solution was added, and the total volume was adjusted to 300 µL using cell staining buffer for flow cytometry analysis. The stained cells were transferred to a flow cytometry tube for detection. The flow cytometer was configured with an excitation wavelength of 488 nm to detect FITC fluorescence at 515 nm and PI fluorescence at 560 nm. Cells in the lower-left quadrant represented viable cells; those in the upper-left quadrant indicated necrotic cells, those in the upper-right quadrant were late-apoptotic cells, and those in the lower-right quadrant were early-apoptotic cells.

**MTT**

Cells were seeded at a density of 5 × 10^3^ cells per well in a 96-well plate, and 200 μL of PBS was added to the periphery of each well to reduce liquid evaporation. The plate was incubated in an incubator for 12 h to allow the cells to adhere. The original culture medium was discarded, and the cells were divided into control and PF (10, 20, 30, 40, 50, 60, 70, and 80 μmol·L^−1^) groups (five replicates per group). Blank or drug-containing culture medium was added to each sample according to the group assignment. Subsequently, cells in the experimental group were subjected to OGD/R. The supernatant was discarded, and 150 μL of MTT working solution was added to each well in the dark. The cells were then incubated in an incubator for 3 h. The supernatant was discarded, and 150 μL of DMSO was added to each well. The plate was then shaken at 600 r·min^−1^ for 10 min. The absorbance (570 nm/650 nm) of cells was immediately measured using a microplate reader. Cell viability was calculated as viability = [average absorbance (drug) − average absorbance (blank)]/[average absorbance (no drug) − average absorbance (blank)] × 100.

**Molecular docking**

The chemical structures of the compounds were drawn using ChemDraw v23.0 and subsequently converted to the “mol2” format to create datasets for molecular docking. Three-dimensional structures of core target proteins were retrieved from the Protein Data Bank (https://www.rcsb.org) (accessed on 25 November 2024). Using PyMOL 3.0.4 and AutoDockTools 1.5.7, the receptor proteins were prepared by removing water molecules and non-essential residues, followed by the addition of hydrogen atoms. The processed proteins were then saved in the “pdbqt” format for use as receptors. Molecular docking was performed using AutoDock Vina, and the resulting docking poses were visualized and analyzed using PyMOL 3.0.4 and LigPlot+ v2.2.5.

**MD simulation**

MD simulations were conducted using GROMACS 2022.2. The force field parameters were generated with the ‘pdb2gmx’ tool implemented in GROMACS 2022.2. The CHARMM36 force field was applied to the receptor protein, whereas the CGenFF force field was employed for the ligand. During system setup, the complex was solvated using the TIP3P water model, with a minimum distance of 1.0 nm set between the protein atoms and the box edges to ensure adequate solvation. To maintain system electroneutrality and approximate physiological ionic strength, Na^+^ and Cl^−^ ions were added using the ‘gmx genion’ tool to a final concentration of 0.15 M NaCl. Long-range electrostatic interactions were treated with the Particle Mesh Ewald method, applying a real-space cutoff of 1.0 nm. All covalent bonds involving hydrogen atoms were constrained using the LINCS algorithm. Before the production MD runs, the system underwent energy minimization, consisting of 3000 steps of the steepest descent method followed by 2000 steps of the conjugate gradient method. The simulations were performed under the NPT ensemble at a constant temperature of 310 K, maintained using a Nosé–Hoover thermostat with a coupling constant of 0.1 ps, and at a constant pressure of 1 bar regulated by a Parrinello–Rahman barostat with a coupling constant of 2.0 ps. The total simulation time was set to 100 ns, with an integration time step of 2 fs. Throughout the trajectories, the GROMACS 2022.2 tools ‘gmx rmsd’, ‘gmx rmsf’, ‘gmx hbond’, ‘gmx gyrate’, ‘gmx sasa’, and ‘gmx sham’ were used to compute the RMSD, RMSF, number of hydrogen bonds, Rg, and SASA, respectively, to evaluate system stability, conformational changes, and solvent effects.

**Network pharmacology**

The canonical SMILES of PF was obtained from PubChem. The targets of PF were identified using SwissTargetPrediction. The SMILES number of PF was entered into Index-Calcnet-TargetNet to obtain target proteins, which were converted into gene targets in UniProt, followed by merging and deduplication with the previous targets. The disease targets of CIRI were retrieved and screened using GeneCards and OMIM, followed by merging and deduplication. Venny 2.1.0 was used to screen the intersection targets of PF and CIRI and create a Venn diagram. The intersection targets were imported into STRING for gene verification. Cytoscape 3.9.1 was used to import the .tsv file and construct the PPI network. Finally, DAVID was used to import the intersection targets. BP, CC, MM, and KEGG files were downloaded, processed, and imported into Metascape and the Microbioinformatics platform for GO enrichment and KEGG pathway analyses.

**OGD/R**

After reaching 85% confluence, SH-SY5Y and HCMEC/D3 cells were seeded into six-well plates at a density of 3 × 10^5^ cells per well and allowed to attach for 24 h. After treatment with PF for 24 h, cells in the model and PF groups were placed in serum- and glucose-free DMEM and transferred to a tri-gas incubator at 37 °C. The OGD duration was counted from when the internal O_2_ concentration dropped to 1.0%, and the exposure time was cell-type-dependent: SH-SY5Y cells were maintained in the conditions for 2 h and 15 min, whereas HCMEC/D3 cells were exposed for 3 h. After OGD, the medium was discarded, 10% complete medium was added, and cells were incubated in a normoxic incubator with 5% CO_2_ for further culture. After 24 h of incubation in glucose-containing medium and reoxygenation, the cells were collected for subsequent experimentation. Control cells were cultured in normal complete medium under normoxic conditions throughout the experiment.

**RNA extraction, library preparation, and sequencing**

Total RNA was isolated from specified biological materials using TRIzol^®^ reagent (Thermo Fisher Scientific, Waltham, MA, USA, Cat# 15596026) following the manufacturer’s protocol. To eliminate potential DNA contamination, RNA samples were treated with DNase I (New England Biolabs, Ipswich, MA, USA, Cat# M0303L). RNA purity was assessed by measuring the absorbance ratio at 260 nm/280 nm using a Nanodrop™ OneC spectrophotometer (Thermo Fisher Scientific). RNA integrity was verified using the LabChip^®^ GX Touch system (Revvity, Waltham, MA, USA). The RNA concentration was quantified using a Qubit^®^ 3.0 fluorometer with the Qubit™ RNA Broad Range Assay Kit (Thermo Fisher Scientific, Cat# Q10210).

The KC™ Digital mRNA Library Prep Kit (Kangce Bio, Wuhan, China) was used for RNA sequencing and library preparation according to the manufacturer’s instructions. This protocol incorporates unique molecular identifiers into cDNA molecules to correct biases and errors introduced during PCR amplification and sequencing. Libraries were generated by enriching 200–500 bp PCR fragments. After quantification, libraries were sequenced on a NovaSeq X Plus platform (Illumina, San Diego, CA, USA) using a paired-end 150 bp sequencing mode, generating approximately 6 Gb of raw data per sample.

Raw sequencing reads were subjected to quality control using fastp (version 0.23.0) [@fastp]. Clean reads were aligned to the GRCh38_release101 reference genome. Gene-level read counts were normalized and expressed as reads per kilobase per million mapped reads.

Differential expression analysis was performed using the criteria of |log_2_ fold change| ≥ 1 and adjusted false discovery rate *p*-value < 0.05. The Benjamini–Hochberg method was applied for multiple testing correction. Three biological replicates were included per experimental group.

**Meta-analysis and data visualization**

Gene targets identified through network pharmacology and DEGs from sequencing data were intersected and analyzed for functional enrichment using the Metascape platform. To validate network pharmacology results, GO enrichment (BPs, CCs, and MFs) and KEGG pathway analyses were performed. Chord diagrams and Sankey bubble plots were subsequently generated using the MicrobioMed platform (www.bioinformatics.com.cn) (accessed on 25 October 2024) to visualize enrichment relationships and pathway associations.

**Cell co-culture assay**

When the confluence of HCMEC/D3 cells reached 90%, conditioned medium (CM) was prepared as follows: HCMEC/D3 cells were first pretreated with PF (40 μM) for 24 h, before the drug-containing culture medium was discarded; cells were then washed twice with PBS and switched to a glucose- and serum-free culture medium for OGD treatment (3 h and 15 min) before the medium was replaced with complete culture medium for reoxygenation (R) treatment for 24 h. Subsequently, the supernatant was collected, centrifuged at 1000 rpm for 4 min, and then filtered through a 0.22 μm sterile syringe filter. The resulting CM was then applied to SH-SY5Y cells in three experimental groups: a control, a CM-only group, and a CM + inhibitor group. Working solutions for SH-SY5Y cell treatment were prepared using fresh complete culture medium, complete culture medium containing 10% CM, or complete culture medium supplemented with 10% CM and the PI3K inhibitor LY294002 (20 μM, added simultaneously with CM). These working solutions were then applied to SH-SY5Y cells for 24 h, after which the apoptosis rate was determined by flow cytometry using the Annexin V-FITC/PI double staining method.

**Statistical analysis**

Data are presented as means ± standard deviation. Statistical analyses were performed using GraphPad Prism (version 8.0.2, GraphPad Software, San Diego, CA, USA). Normality was assessed using the Shapiro–Wilk test, and homogeneity of variance was evaluated using Levene’s test. For data meeting the assumptions of normality and homogeneity of variance, differences among groups were analyzed by one-way analysis of variance followed by Tukey’s honestly significant difference post hoc test. When variances were unequal, Welch’s analysis of variance followed by the Games–Howell test was performed. For non-normally distributed data, the Kruskal–Wallis H test was used, followed by Dunn’s post hoc test. All tests were two-tailed, and statistical significance was defined as *p* < 0.05. Each group consisted of three biological replicates. Each sample was measured in triplicate as technical replicates, and the means of the technical replicates were calculated.

All web addresses mentioned in the Materials and Methods are compiled in Table 2.

## 5. Conclusions

This study employed network pharmacology, transcriptomic analysis, molecular docking and MD simulations, combined with SH-SY5Y neuronal and HCMEC/D3 human cerebral microvascular endothelial cell OGD/R cell models, neuron–endothelial cell co-culture systems, pathway-specific inhibition experiments, and Western blot assays, to preliminarily investigate the potential molecular mechanisms by which PF alleviates CIRI. PF was found to exert neuroprotective effects through the VEGF/PI3K-AKT/mTOR pathway by directly activating neurons and indirectly modulating the endothelium-mediated microenvironment, deepening our understanding of its neuroprotective effects. However, these results were primarily derived from computational predictions and in vitro cell models because the OGD/R model cannot replicate the complex pathological environment of the BBB and neurovascular unit in vivo. Further validation using in vivo animal models is still required.

Overall, these findings suggest that PF attenuates OGD/R-induced neuronal dysfunction and apoptosis by activating the PI3K/VEGF-AKT/mTOR pathway, providing cellular-level evidence for its neuroprotective effects. The extension of this mechanism toward clinical application still needs to be established through in vivo animal models and preclinical studies.

## Figures and Tables

**Table 1:** Table of molecular docking binding energies.

Molecular Name	Binding Energy
VEGF-A (PDB: 5DN2)	−8.4 kcal/mol
AKT (PDB: 6S9W)	−5.5 kcal/mol
mTOR (PDB: 9F42)	−9.6 kcal/mol

**Table 2:** Websites used in the experiments.

Web Tool/Software/Experimental Platforms	Uniform Resource Locator
PDB	https://www.rcsb.org
STRING	https://string-db.org
PubChem	https://pubchem.ncbi.nlm.nih.gov
SwissTargetPrediction	http://www.swisstargetprediction.ch
UniProt	https://www.uniprot.org
GeneCards	https://www.genecards.org
OMIM	https://www.omim.org
DAVID	https://davidbioinformatics.nih.gov/
Metascape	https://metascape.org
Venny 2.1.0	https://bioinfogp.cnb.csic.es/tools/venny (accessed on 15 October 2024)
MicrobioMed	www.bioinformatics.com.cn
Cytoscape 3.9.1	
PyMOL 3.0.4	
AutoDockTools 1.5.7	
AutoDockTools Vina 1.2.5	
Gromacs 2022	
ChemDraw	
NovaSeq X Plus (Illumina)	
Nanodrop™ OneC	
LabChip^®^ GX Touch	

## Data Availability

The original contributions presented in this study are included in the article. Further inquiries can be directed to the corresponding authors.

## Abbreviations

The following abbreviations are used in this manuscript:

AKT	Protein Kinase B
BP	Biological Process
CC	Cellular Component
CIRI	Cerebral Ischemia–Reperfusion Injury
DEG	Differentially Expressed Gene
DMEM	Dulbecco’s Modified Eagle Medium
DMSO	Dimethyl Sulfoxide
ECM	Extracellular Matrix
GO	Gene Ontology
KEGG	Kyoto Encyclopedia of Genes and Genomes
MF	Molecular Function
mTOR	Mammalian Target of Rapamycin
MTT	Methyl Thiazolyl Tetrazolium
OGD/R	Oxygen Glucose Deprivation/Reoxygenation
PPI	Protein–Protein Interaction
PF	Paeoniflorin
PI3K	Phosphatidylinositol 3-Kinase
VEGF	Vascular Endothelial Growth Factor
VEGFA	Vascular Endothelial Growth Factor A
WB	Western Blotting

## Appendix A

**Table A1:** Table of the top nine molecular docking results for VEGF-A binding with the PF compound.

Mode	Affinity (kcal/mol)	Dist from Best Mode Rmsd l.b.	Dist from Best Mode Rmsd u.b.
1	−8.4	0.000	0.000
2	−8.3	1.778	5.440
3	−7.6	2.028	8.277
4	−7.6	2.118	7.250
5	−7.6	2.642	5.624
6	−7.5	2.208	6.373
7	−7.1	2.135	7.656
8	−7.0	21.031	26.480
9	−7.0	29.616	33.010

**Table A2:** Table of the top nine molecular docking results for AKT binding with the PF compound.

Mode	Affinity (kcal/mol)	Dist from Best Mode Rmsd l.b.	Dist from Best Mode Rmsd u.b.
1	−5.5	0.000	0.000
2	−5.5	16.671	19.445
3	−5.0	11.789	16.144
4	−5.0	15.377	18.227
5	−4.9	5.044	7.950
6	−4.6	1.522	1.925
7	−4.5	6.174	9.831
8	−4.4	17.292	20.003
9	−4.3	2.471	4.928

**Table A3:** Table of the top nine molecular docking results for mTOR binding with the PF compound.

Mode	Affinity (kcal/mol)	Dist from Best Mode Rmsd l.b.	Dist from Best Mode Rmsd u.b.
1	−9.6	0.000	0.000
2	−8.4	2.396	4.755
3	−8.1	3.597	7.347
4	−7.5	62.603	65.511
5	−7.3	3.577	8.715
6	−7.2	50.065	52.392
7	−7.0	34.015	36.866
8	−7.0	92.620	96.656
9	−7.0	55.063	59.184
